# The developing premature infant gut microbiome is a major factor shaping the microbiome of neonatal intensive care unit rooms

**DOI:** 10.1186/s40168-018-0493-5

**Published:** 2018-06-20

**Authors:** Brandon Brooks, Matthew R. Olm, Brian A. Firek, Robyn Baker, David Geller-McGrath, Sophia R. Reimer, Karina R. Soenjoyo, Jennifer S. Yip, Dylan Dahan, Brian C. Thomas, Michael J. Morowitz, Jillian F. Banfield

**Affiliations:** 1 Department of Plant and Microbial Biology, University of California, Berkeley, CA USA; 2 University of Pittsburgh School of Medicine, Pittsburgh, PA USA; 3 Division of Newborn Medicine, Children’s Hospital of Pittsburgh of UPMC, Pittsburgh, PA USA; 4 Department of Earth and Planetary Sciences, University of California, Berkeley, CA USA; 5 Department of Biology, Bard College, Annandale-on-Hudson, NY USA; 6 Present address: Department of Microbiology and Immunology, Stanford University School of Medicine, Stanford, CA USA

**Keywords:** Infant gut, Microbiome, Built environment, Neonatal intensive care unit

## Abstract

**Background:**

The neonatal intensive care unit (NICU) contains a unique cohort of patients with underdeveloped immune systems and nascent microbiome communities. Patients often spend several months in the same room, and it has been previously shown that the gut microbiomes of these infants often resemble the microbes found in the NICU. Little is known, however, about the identity, persistence, and absolute abundance of NICU room-associated bacteria over long stretches of time. Here, we couple droplet digital PCR (ddPCR), 16S rRNA gene surveys, and recently published metagenomics data from infant gut samples to infer the extent to which the NICU microbiome is shaped by its room occupants.

**Results:**

Over 2832 swabs, wipes, and air samples were collected from 16 private-style NICU rooms housing very low birth weight (< 1500 g), premature (< 31 weeks’ gestation) infants. For each infant, room samples were collected daily, Monday through Friday, for 1 month. The first samples from the first infant and the last samples from the last infant were collected 383 days apart. Twenty-two NICU locations spanning room surfaces, hands, electronics, sink basins, and air were collected. Results point to an incredibly simple room community where 5–10 taxa, mostly skin-associated, account for over 50% of the amplicon reads. Biomass estimates reveal four to five orders of magnitude difference between the least to the most dense microbial communities, air, and sink basins, respectively. Biomass trends from bioaerosol samples and petri dish dust collectors suggest occupancy to be a main driver of suspended biological particles within the NICU. Using a machine learning algorithm to classify the origin of room samples, we show that each room has a unique microbial fingerprint. Several important taxa driving this model were dominant gut colonizers of infants housed within each room.

**Conclusions:**

Despite regular cleaning of hospital surfaces, bacterial biomass was detectable at varying densities. A room-specific microbiome signature was detected, suggesting microbes seeding NICU surfaces are sourced from reservoirs within the room and that these reservoirs contain actively dividing cells. Collectively, the data suggests that hospitalized infants, in combination with their caregivers, shape the microbiome of NICU rooms.

**Electronic supplementary material:**

The online version of this article (10.1186/s40168-018-0493-5) contains supplementary material, which is available to authorized users.

## Background

Hospital acquired infections (HAIs) remain a major problem in the USA. One out of every 25 patients will experience a HAI, costing the USA approximately $30 billion per year [1]. Infants hospitalized in the neonatal intensive care units (NICU) are particularly susceptible to infection due to their underdeveloped immune systems [2, 3]. To protect against infection, infants are often prescribed antibiotics during the first week of life. In fact, antibiotics are three of the six most commonly administered medications in the NICU [4]. This treatment likely kills microbes acquired during the birthing process [5] and promotes a categorically different colonization pattern in preterm infants relative to full-term infants [6]. Preterm infants are often colonized by ESKAPE organisms (*Enterococcus spp.*, *Staphylococcus aureus*, *Klebsiella spp.*, *Acinetobacter spp.*, *Pseudomonas aeruginosa*, and other *Enterobacteriaceae*), which are also the most frequent cause of nosocomial infections [7]. The relatively sterile preterm infant gut microbiome and the high frequency at which infants are colonized by hospital-associated microbes create a valuable study setting to better understand how the room microbiome is shaped by its occupants. Here, we conducted an experiment to quantify and characterize NICU room microbes to enable comparison with microbiomes that develop in the premature infant gut.

The source of early-stage gut colonizers in preterm infants has been explored to some extent [8, 9]. In a pilot study, we tracked two infants over the first month of life, collecting samples from room surfaces and infant fecal samples [10]. Using an amplicon-EMIRGE approach, which allows for recovery of full-length 16S rRNA genes (~ 1500 b) [11], as opposed to the more common hypervariable region approach (~ 150–400 b), we detected the same sequences in room samples before they were detected in gut samples. In a much higher resolution genome-resolved metagenomics study, we recently showed evidence for the presence of some infant gut-associated strains in the NICU room environment and for exchange of those strains between infant and room environments [12].

Recent genomic studies have shown that the vast majority of strains in the premature infant gut are not shared among infants [5]. Nearly 150 strains were recovered from 10 infants’ fecal samples, and only four of these were shared. These samples were collected within a month of each other, suggesting that a multitude of strains are available in the NICU at any given point in time, and only a few strains may be widespread, a conclusion supported by the more recent research [13]. However, a few strains were identified in infant fecal samples collected years apart from different infants housed the same NICU [14]. These were referred to as “persister” strains.

A recent study identified 794 antibiotic resistance genes in preterm infant stool samples, 79% which had not previously been classified as associated with resistance [15]. It is possible that these genes provide a competitive advantage for survival in the highly cleaned room environment [16]. However, in our prior work, we found that persister strains, which we infer have a room reservoir, were not found to differ significantly in virulence, antibiotic resistance, or metabolism from non-persister strains.

An important question from the perspective of HAI and microbiome establishment of hospitalized premature infants relates to the diversity and biomass distributions over room environments. To address this knowledge gap, we conducted a study with 16 infants, whose rooms were sampled Monday through Friday from 22 room locations. We performed droplet digital PCR (ddPCR) on all room samples to directly quantify biomass (2832 samples in total) to determine how biomass varies in the NICU with additional quantification of negative controls. Overall, the findings provide new information about the NICU microbiome and its relationship to room occupant microbiomes.

## Methods

### Sample collection

Eighteen infants were enrolled in the study based on the criteria that they were in < 33 weeks gestation and were housed in the same physical location within the NICU during the first month of life. Two infants were excluded due to medical complications. Samples were collected Monday through Friday for days of life (DOL) 5–28. Fecal samples were collected from infant diapers and were stored at − 20 °C within 10 min of collection for short-term storage. Shortly after collection, samples were archived and transferred to a − 80 °C freezer for long-term storage until DNA extraction. All samples were collected after signed guardian consent was obtained, as outlined in our protocol to the ethical research board of the University of Pittsburgh (IRB PRO12100487). This consent included sample collection permissions and consent to publish study findings.

All samples were obtained from a private-style NICU at Magee-Womens Hospital of the University of Pittsburgh Medical Center. Each infant was housed within an incubator (Giraffe Omnibed) with one incubator per room. Rooms measured 4.6 × 2.9 × 2.4 m (L × W × H), and each had similar design and equipment. A detailed description of the hospital floor plan is available in previously published studies of the same NICU [17, 18]. Twenty-two of the most frequently touched surfaces were determined by visual observation and healthcare provider interviews in the weeks leading up to sample collection. Microbial cells were removed from most surfaces using nylon FLOQSwabs (Copan Diagnostics, Brescia, Italy) and a sampling buffer of 0.15 M NaCl and 0.1% Tween20. Samples were collected by one research nurse to ensure consistent sampling technique. Ten square centimeters of each surface was sampled or, for smaller surfaces, the entire surface itself (e.g., isolette knobs and sink basin drain grill). Wipe samples were collected from the floor and exterior top of the isolette using Texwipe TX1086 wipes (Texwipe, Kernersville, NC, USA). Before collecting each wipe sample, the collector would put on latex examination gloves and clean these gloves with an isopropanol wipe. The wiped surface area was approximately 48 cm^2^ or, for smaller surfaces, the entire surface itself (e.g., isolette top). A wipe was also used to collect microbial cells at the exterior facet of the heating, ventilation and air conditioning (HVAC) system. The wipe was suspended via airflow on the exterior (upstream) face of the MERVE 8 pleated filter, the zone in which supply and return air are mixed before thermal and humidity treatment of the airstream for 4 days. Features of the HVAC system are described in detail in a recently published paper [17].

Air samples were collected using the NIOSH two-stage bioaerosol cyclone 251 sampler [19] and a suspended petri dish method [20]. The NIOSH sampler collected samples continuously Monday through Friday, comprising approximately 96 h of sampling at 3.5 L/min (total volume sampled = 20 m^3^). Petri dish samples were suspended approximately 1 m below the drop ceiling in the corner of the room that was the furthest away from the sink. These samplers were maintained in place for the duration of the infant’s stay. Petri dish “cooler” samples are plates that were taped to the top of a cooler which collected abiotic aerosol data [17]. At the end of the sample collection period, all samples were placed in a sterile transport tube and stored within 10 min at − 80 °C until further processing.

### DNA extraction and PCR amplification

DNA was extracted using either the MO BIO PowerSoil DNA Isolation kit (single tube extractions) or PowerSoil-htp 96-well DNA Isolation kit (MoBio Laboratories, Carlsbad, CA, USA). For DNA extracted from feces with the 96-well kit, fecal samples were kept frozen on dry ice and added to individual wells of the bead plate and stored at − 80 °C until extraction. In the day of extraction, Bead Solution and Solution C1 were added and the plates were incubated at 65 °C for 10 min. The plates were shaken on a Retsch Oscillating Mill MM400 with 96-well plate adaptors for 10 min at speed 20. The plates were rotated 180° and shaken again for 10 min at speed 20. All remaining steps followed the manufacturer’s centrifugation protocol. For swab samples, the heads were snapped at the perforation into the wells of the bead plate and stored at − 80 °C. In the day of extraction, the Bead Solution and Solution C1 were added and the plates were incubated at 65 °C for 10 min. The plates were shaken on a Retsch Oscillating Mill MM400 with 96-well plate adaptors for 5 min at speed 20. The plates were rotated 180° and shaken again for 5 min at speed 20. The solution C2 and C3 steps were combined (200 μL of each added) to improve DNA yield. All remaining steps followed the manufacturer’s centrifugation protocol.

Wipe samples were stored in a sterile 250-mL tissue culture flask upon collection and thawed on ice before extraction. Cells were dislodged from wipes in a protocol adapted from Yamamoto et al. [21]. Briefly, 150 mL of dislodging buffer was poured into a flask (1X PBS, 0.04% Tween 80, passed through a 0.2-μm filter), and the flask was shaken vigorously for 1 min. Supernatant was then decanted into a 250-mL disposable filter funnel with a pore size of 0.2 μm (Thermo Scientific, Waltham, MA, USA), and the filter was then placed in a MoBio PowerWater extraction tube. PowerWater extraction followed manufacturer recommendations.

Droplet digital PCR (ddPCR) was adapted from a method previously published on quantification of 16S rRNA templates in infant fecal samples [5]. The only deviation from the previous method was that a diluted gDNA template of 1:10 instead of 1:1000 was utilized. Both MiSeq library preparation and ddPCR were performed in a 96-well plate format. Each plate had three no template PCR controls, one no template extraction control, and three positive controls containing varying concentrations of purified *E. coli* gDNA. Counts from the negative control types were averaged across types, and the highest was used to correct for contaminant counts in sample data.

### Sequencing preparation and sequencing

Genomic DNA from room samples were subjected to 16S rRNA V3-4 MiSeq library preparation which included dual-barcoded multiplexing with a heterogeneity spacer for higher sequence quality [22]. Two microliters of 5× concentrated gDNA template was used in the reaction and run at 35 cycles. Amplicons were purified using the Just-a-Plate PCR normalization and purification kit (Charm Biotech, San Diego, CA, USA). Equal amounts of each sample were sent to the University of California Davis DNA Technologies Core Facility (http://dnatech.genomecenter.ucdavis.edu) and run on a MiSeq with v3 300PE chemistry.

Illumina library construction for infant fecal samples followed standard protocols at University of California QB3 Vincent J. Coates Genomics Sequencing Core Facility (http://qb3.berkeley.edu/gsl/). Briefly, gDNA was sheared using a Covaris to approximately 600 and 1000 bp. Wafergen’s PrepX DNA library prep kits were used in conjunction with the Apollo324 robot following factory recommendations (Integenx). Thirteen cycles of PCR were used during library construction. Libraries were added at 12 samples per lane, in equimolar amounts, to the Illumina HiSeq 2500 platform. Paired-end sequences were obtained with 150 cycles and the data processed with Casava version 1.8.2. Raw read data were deposited in the NCBI Short Read Archive (Bioproject PRJNA376566, SRA SRR5405607 to SRR5406014).

### 16S rRNA gene amplicon data processing

The LotuS 1.562 pipeline in short amplicon mode was used for quality filtering, demultiplexing, and OTU picking [23]. LotuS was run with the following command line options: “-refDB SLV,GG -highmem 1 -p miseq -keepUnclassified 1 -simBasedTaxo lambda -threads 10.” OTU data was rarefied to 1000 sequences per sample, without replacement, unless explicitly stated. OTU table and LotuS log files are available on figshare [12, 24].

### Metagenomic data from infant gut samples

For comparative purposes, this study made use of previously published infant metagenomic data from 290 fecal samples collected from infants housed in the NICU rooms studied here (~ 800 Gb of 150 bp paired-end reads). Methods for data analysis and infant metadata (e.g., gestational age, birth weight, vaginal or cesarean delivery) are described within this publication [13].

### Statistics and data visualizations

To determine if infant room samples have specific bacterial communities, we adopted a support vector machine (SVM) approach previously implemented for a similar question (i.e., do offices within a given city have bacterial communities that look more like the communities in other offices in the same city than those in offices in other cities) [25]. Samples not directly within an infant’s room (i.e., hallway, HVAC, and nursing station samples), environments with less than 50 samples per infant, and taxa less than 0.001% were removed from the analysis. Eighty percent of the data was used in the training dataset (779 samples, 1700 OTU predictors), and tuning the SVM model was conducted using the R package Caret [26] with five-fold cross validation. Caret’s varImp function was used to assess feature importance, which calculates the area under the receiver operating characteristic curve. Manipulation of compositional data was performed using phyloseq [27], and visualizations were done using ggplot2 [28].

## Results

### Sequencing summary and contamination removal

In total, 2832 room samples were processed through a MiSeq library preparation protocol. After quality filtering and demultiplexing, 84,939,529 read pairs were generated. These reads were clustered into 18,093 OTUs. Using a ratio OTU (ROTU) method that leverages biomass quantification and sequencing of negative controls [29], 269 OTUs and 925 samples were removed from the dataset when using an ROTU threshold of 0.001. A second in silico contamination cleaning method was applied [30], which removed an additional 323 OTUs and one sample. In total, approximately 3% of generated OTUs and 33% of samples present too weak of a signal to confidently distinguish them from negative control signatures.

### Biomass and taxonomic variation across petri dish replicates

Biological and technical replicates performed for petri dish plates established the reproducibility of extraction of DNA from petri dish swabs and provided evidence for highly reproducible ddPCR measurements (Additional file 1). The highest standard deviation in ddPCR values for biological replicates in a single room was 106,760 copies/sample (infant 6’s petri plates; mean = 99,677), and for technical replicates, the largest standard deviation was 15,534 copies/sample (infant 12’s petri plates, mean = 81,044). The lowest standard deviation for biological replicates was 1981 copies/sample (infant 1’s petri plates, mean = 13,785) and 737 copies/sample for technical replicates (infant 11’s petri plates, mean = 32,396). Overall, this equates to a reproducibility range of 2.69 to 6.87× more reproducibility across technical ddPCR runs relative to biological replicates, with an average reproducibility ratio of 5.37× better for technical replicates.

### Biomass varies significantly across sample types

**Fig. 1:**
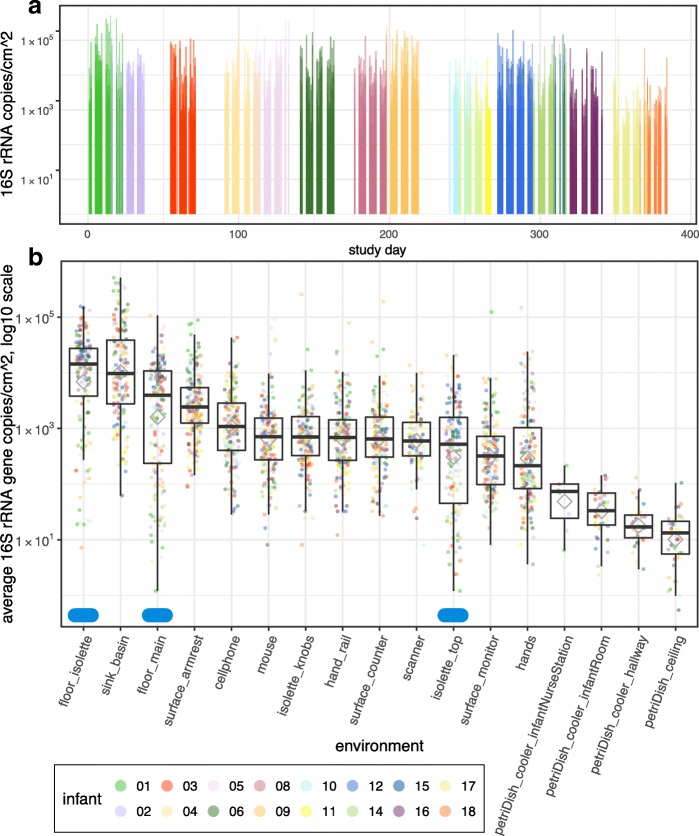
Biomass varies by four to five orders of magnitude in a NICU. 16S rRNA gene template copy number was quantified via ddPCR. **a** Biomass was averaged across all swab and wipe samples for each sampling day and plotted on a timeline to visualize variation in biomass over the sampling campaign. **b** Each dot reflects the average across triplicate runs. Gray diamonds represent averages per environment. Blue ellipses along the *x*-axis represent samples collected using a wipe method. All other samples were collected with swabs or using a petri plate to collect settled dust (noted in label). All counts are normalized to represent 1 day of collection

16S rRNA gene copies were quantified for 2883 samples using ddPCR and showed day-to-day variation ranging from approximately 4 to 33,000 16S rRNA copies/cm^2^ (Fig. 1a). Samples from the HVAC system had the highest biomass of all types, and bioaerosol samples had the lowest (Additional file 2 a and b). Sinks had the highest biomass of the swabbed samples and hands had the lowest average median template count (Fig. 1b). Petri dishes suspended from the ceiling had the lowest biomass relative to other passive dust collectors, whereas the nurse’s station dishes contained the highest bacterial load. The infant room consistently had higher template counts than the hallway bioaerosol samples. Overall, the median biomass varied over four orders of magnitude across all sample types.

### Skin-associated taxa dominate the NICU surface environment

**Fig. 2:**
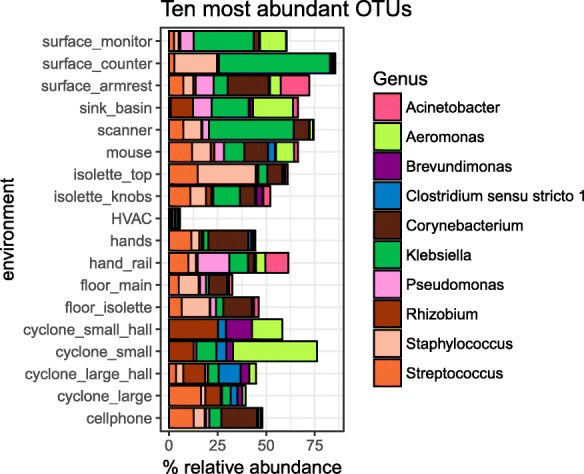
Top 10 NICU OTUs comprise > 50% of NICU taxa. Amplicon data from a 16S rRNA gene V3-4 workflow is plotted for each environment. Only the top 10 OTUs, determined from averages across all samples, are plotted. Each OTU is colored by its genus-level classification

The microbial communities in most NICU environments were highly uneven and were dominated by 5–10 OTUs (Fig. 2). Forty-one percent and 55% of all amplicon reads belong to the top 5 and 10 OTUs in the NICU, respectively (Fig. 2 and Table 1). Most of these taxa are human-associated with many commonly associated with the skin (*Corynebacterium*), mouth (*Streptococcus*), or nose (*Staphylococcus*). SourceTracker v1.0.1 [31] was run using skin, oral, and fecal samples from the American Gut project as the putative source database with NICU samples labeled as “sink” samples. Skin was the most likely contributor to taxa in the NICU, accounting for upwards of 50% of the most probable sources, followed by oral and fecal samples (Additional file 3).

**Table 1:** Top 10 OTUs in the NICU

OTU	Kingdom	Phylum	Class	Order	Family	Genus	Species	Rel. abund. (%)
OTU_5	Bacteria	Proteobacteria	Gammaproteobacteria	Enterobacteriales	Enterobacteriaceae	Klebsiella	?	12.9
OTU_6	Bacteria	Firmicutes	Bacilli	Bacillales	Staphylococcaceae	Staphylococcus	?	7.3
OTU_4	Bacteria	Actinobacteria	Actinobacteria	Corynebacteriales	Corynebacteriaceae	Corynebacterium	?	7.1
OTU_7	Bacteria	Firmicutes	Bacilli	Lactobacillales	Streptococcaceae	Streptococcus	?	6.9
OTU_9	Bacteria	Proteobacteria	Gammaproteobacteria	Aeromonadales	Aeromonadaceae	Aeromonas	?	6.9
OTU_10	Bacteria	Proteobacteria	Alphaproteobacteria	Rhizobiales	Rhizobiaceae	Rhizobium	?	4.5
OTU_8	Bacteria	Proteobacteria	Gammaproteobacteria	Pseudomonadales	Pseudomonadaceae	Pseudomonas	?	3.7
OTU_11	Bacteria	Proteobacteria	Gammaproteobacteria	Pseudomonadales	Moraxellaceae	Acinetobacter	?	2.3
OTU_30	Bacteria	Firmicutes	Clostridia	Clostridiales	Clostridiaceae 1	Clostridium sensu stricto 1	?	1.9
OTU_32	Bacteria	Proteobacteria	Alphaproteobacteria	Caulobacterales	Caulobacteraceae	Brevundimonas	?	1.8

Samples collected from the HVAC system had the highest bacterial diversity with 405 OTUs on average per sample, whereas bioaerosol samples had the lowest, with 13 (Additional file 4a). The HVAC samples had the highest Shannon community evenness, followed by floor wipes, and the bioaerosol samples had the lowest Shannon diversity (Additional file 4b). Thus, overall, the HVAC had highly even consortia with high diversity. This is expected due to the way that the HVAC sample was collected, with metric tons of air passing through the collection wipe before sequencing [17]. The NICU room air was also found to have low biomass and low diversity, with strong dominance by members of the *Aeromonadaceae* in the small size fraction and *Streptococcaceae*, *Rhizobiaceae*, and *Clostridiaceae* in the large size fraction.

All touched surfaces had similar numbers of OTUs per sample, although the surface monitors showed the most unevenness (Additional file 4). These surfaces were dominated by similar groups of microbes. Although many touched surfaces were associated with skin-associated bacteria, a gut-associated *Klebsiella* OTU also dominated environments such as the surface monitors, counter tops, and scanners (Fig. 2). In contrast, the sink basins had comparatively low numbers of OTUs per sample (Additional file 4a), in part due to the high dominance by four bacterial groups (Fig. 2).

### Biomass suggests growth patterns in sink basins

**Fig. 3:**
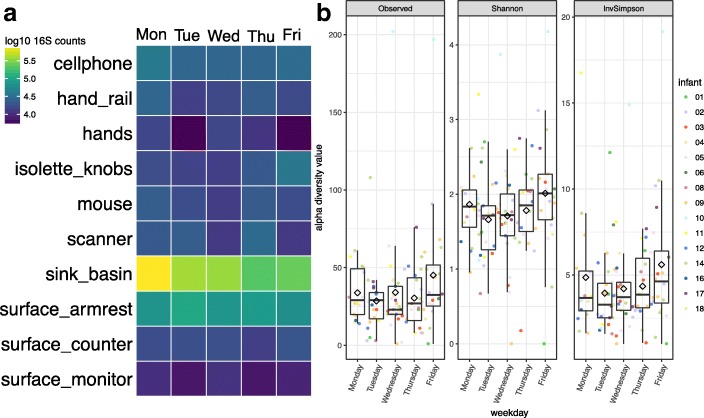
Growth detected in NICU sink samples. 16S rRNA gene template copy number was quantified via ddPCR. Average copy number was averaged for each weekday and swabbed environment and displayed in this heatmap (**a**). 16S rRNA gene amplicon data was used to calculate number of OTUs, Shannon, and inverse Simpson diversity metrics for sink basin samples (**b**). Black diamonds represent averages per weekday

A range of 29 to 38 sink basin samples per weekday were collected from 14 unique sink basins. When comparing biomass trends across days (Fig. 3a), a distinct pattern of decreasing biomass is apparent in sink samples relative to other swabbed environments. In comparing Shannon diversity across weekdays (Fig. 3b), bacterial diversity in Tuesday versus Friday samples were the most distinct, whereas biomass was most different in Monday versus Thursday samples (Wilcoxon rank sum, Bonferroni adjusted *p* = 0.47 and 0.012, respectively). Sink basins were cleaned approximately every 24 h, but less frequently on the weekends, so the elevated biomass at the beginning of the week may be due to regrowth of sink-adapted taxa throughout the weekend (e.g., *Rhizobiaceae*, *Pseudomonas*, *Aeromonas*, and *Enterobacteriaceae*). The increase in Shannon diversity from Tuesday to Friday strengthens this inference.

### NICU rooms harbor a unique microbial signature

**Fig. 4:**
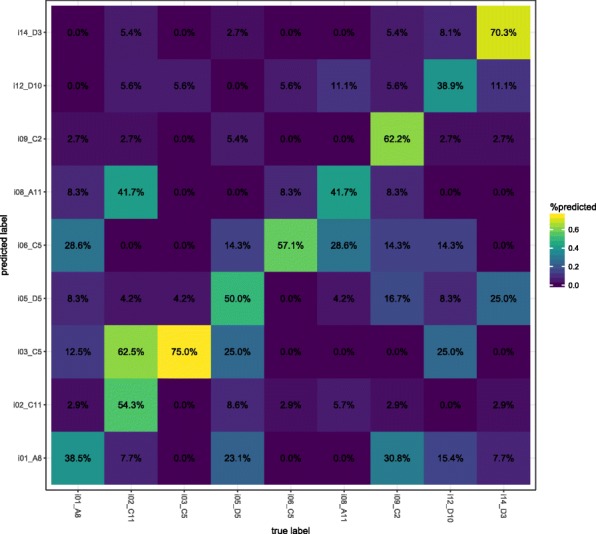
NICU rooms have a unique microbial signature. 16S rRNA gene amplicon data was split into training, test, and validation sets to train, test, and validate a support vector machine classifier. The confusion matrix plots the accuracy of our model on the validation dataset. Percentages note the number of times a sample was predicted to belong to a room-infant pairing divided the total number of samples for that room-infant pairing. The heat coloring is based on shown percentages

Using a support vector machine (SVM) classifier with a linear kernel [25], we determined that each room’s microbiome contained a unique microbial fingerprint. We could predict the room origins with an overall accuracy of 56%, which is 5× better than random chance (Fig. 4). The use of ROTU over a standard pipeline achieved an increase in accuracy of approximately 16%. Typically, the most confusion occurred between samples that were collected at similar times, although infants that had similar gut communities had decreased prediction accuracy (e.g., infants 2, 3, and 8). No misclassification was seen in infants that were housed in the same room at different time periods (i.e., infants 3 and 6), suggesting no room effect over these time scales. Important OTUs driving the SVM model are plotted and listed in Additional file 5 and Table 2. Interestingly, there is an overlap between room-specific OTUs that drive the SVM model and occurrence of these taxa in the gut of infant occupants. For example, the most visible signature in SVM taxa comes from a spike in *Veillonella* in infant 6’s room on DOL 18 (Additional file 5). A major increase of *Veillonella* in infant 6’s gut occurred on DOL 16 (ref http://ggkbase.berkeley.edu/project_groups/human-gut-metagenome-sloan-infants and Additional file 6). The same pattern is seen for infant 8, and in fact, most infants that contain *Veillonella* have strong SVM signals associated with their room. The second strongest signal from the SVM model comes from a *Clostridium* OTU. This group is present in infants 2, 3, and 8’s room samples, and it strongly contributes to the SVM model prediction. All three of these infants have high abundances of *Clostridium*.

**Table 2:** Most important variables to SVM model

OTU	Kingdom	Phylum	Class	Order	Family	Genus	Species
OTU_29	Bacteria	Firmicutes	Clostridia	Clostridiales	Clostridiaceae 1	Clostridium sensu stricto 1	Uncultured organism
OTU_39	Bacteria	Actinobacteria	Actinobacteria	Micrococcales	Micrococcaceae	Rothia	Uncultured organism
OTU_41	Bacteria	Firmicutes	Bacilli	Bacillales	Family XI	Gemella	?
OTU_30	Bacteria	Actinobacteria	Actinobacteria	Micrococcales	Micrococcaceae	Kocuria	?
OTU_45	Bacteria	Actinobacteria	Actinobacteria	Actinomycetales	Actinomycetaceae	Actinomyces	?
OTU_43	Bacteria	Firmicutes	Bacilli	Bacillales	Alicyclobacillaceae	Tumebacillus	Uncultured Firmicutes bacterium
OTU_76	Bacteria	Firmicutes	Clostridia	Clostridiales	Family XI	Peptoniphilus	?
OTU_74	Bacteria	Actinobacteria	Actinobacteria	Actinomycetales	Actinomycetaceae	Actinomyces	Uncultured organism
OTU_28	Bacteria	Firmicutes	Negativicutes	Selenomonadales	Veillonellaceae	Veillonella	Uncultured organism
OTU_66	Bacteria	Firmicutes	Bacilli	Lactobacillales	Streptococcaceae	Streptococcus	?

### Composition of persister taxa in the room echoes infant gut composition

To visualize the distribution of genera with representative strains known to persist in infants over multi-year periods [14, 15], we collapsed each study day and infant pairing by averaging all amplicon abundance data across environments (Additional file 7, “average” panel). In this analysis, the subset of all OTUs that belonged to a persister genus was assigned a distinct color but often one OTU could be distinguished within a genus. However, due to high abundance, we gave OTU_5 (a *Klebsiella*) dedicated coloring. Surprisingly, persister genera account for > 50% of the data at many time points.

Episodes of particularly high persister abundance occurred in rooms housing infants 1, 9, 12, and 16. To better visualize which samples contributed to the averaged data (Additional file 7, “average” panel), we also plotted data for the specific environments for which we had the most samples (armrests and sinks). Both the armrests and sinks are dominated by these groups of organisms during these episodes, but *Staphylococcus* OTUs are much more abundant in armrest samples relative to sinks. Two dominant Pseudomonas OTUs that comprised 70 and 24% of all Pseudomonadaceae (OTU_8 and OTU_15, respectively) were detected throughout the time series but were at very low abundance in armrest samples over long time spans.

### Composition of persister taxa in infant 9

**Fig. 5:**
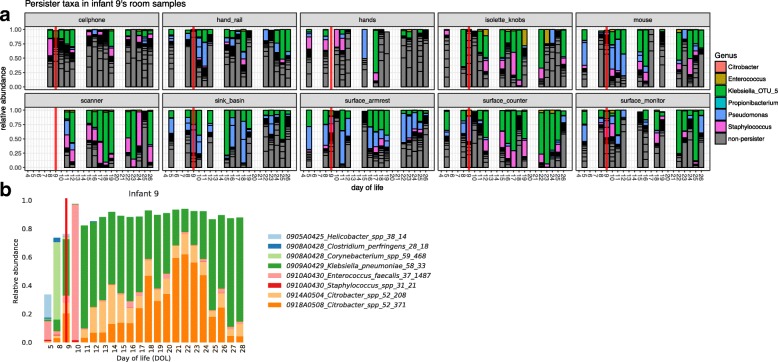
Persister taxa in the room reflect composition of the infant gut. Infant 9’s room amplicons are plotted for each swabbed environment (**a**). Colored are OTUs that belong to a persister lineage. Red lines highlight day of life 9, which coincides with an increase of several *Enterobacteriaceae* taxa in the infant gut (**b**). **b** is the microbial profile for fecal samples generated via genomes recovered from a metagenomics approach

Since the room data for infant 9 had a strong signal for persister groups, we analyzed samples from all environments separately to visualize temporal patterns (Fig. 5a). Persister groups dominated most of infant 9’s room samples, with cellphones having the fewest and scanner and surface counter samples having the most persister groups per sample. The red lines in Fig. 5a highlight the time point where a major increase in relative abundance of *Enterobacteriaceae* taxa occurred in infant 9’s gut (Fig. 5b and Additional file 6). This group is present in multiple room environments prior to the increase, particularly associated with the isolette and armrest. At subsequent time points, this group becomes highly prominent in some room environments (e.g., scanner and surface counter).

OTUs belonging to the persister groups cannot be confidently classified to the species level via 16S rRNA gene sequencing [32], and since *Enterobacteriaceae* dominates the gut of infant 9, we leveraged room and fecal sample context to infer a possible identity for OTU_5. Using OTU_5’s reference sequence as a query, we ran ublast [33] on a database of 16S rRNA genes reassembled from infant 9’s fecal metagenomic samples using the REAGO algorithm [11, 34]. The top hit to our 429-bp query was 99.5% identical (2 mismatches) and came from several of infant 9’s fecal samples. Most of the top hits have the entire 16S rRNA gene recovered from the REAGO assembly (~ 1520 bp). These fecal sequences were searched against the Silva database (SLV_119_SSU) and returned identical, full-length matches to *Klebsiella pneumoniae*. While this is an extrapolation from the V3-4 region, it is possible that OTU_5 in the room is a *Klebsiella* and may be *Klebsiella pneumoniae*, the dominant bacterium colonizing infant 9.

## Discussion

The first question that we aimed to answer in this study related to how biomass varies across a NICU. Using ddPCR to quantify 16S rRNA gene copy number, we show biomass density varies across NICU surfaces by four to five orders of magnitude (Fig. 1). Surprisingly, the floor in front of the infant’s isolette had the highest density of microbes relative to any other environment within the NICU. Naively, it may seem intuitive that the region with the most foot traffic, e.g., the floor at the main entrance of the NICU, would have the highest biomass. While the main entrance floor has a high density, it is significantly lower than the floor in front of the isolette. This finding may be due to the increased occupancy at the isolette versus the main entrance, where occupancy is more transient.

Petri dish data also suggest that higher levels of human activity drive higher amounts of microbial deposition in the room environment. The nursing station has higher petri dish-associated biomass than the infant room, followed by the hallway (Fig. 1). This outcome occurred despite the fact that the infant room and hallway coolers collected dust at the same height (1 m), whereas the nurse station collector was at approximately double the height (1.8 m). As the height above the floor increases, detection of resuspended particles from dust decreases exponentially [35, 36]. This finding suggests that floor dust is not the main source of biological particles accumulated in the petri dishes, but rather, the microbes are human-derived. Greater occupancy or rigor of activity [18] at the nursing station compared to the infant room and hallway likely explains this result.

A recently published study noted a stronger occupancy signal from the occupancy sensors in the infant room compared to the hallway [17]. The occupancy signal directly overlapped with the coarse particle signal (which detected particles > 10 μm in diameter). This signal was interpreted to indicate that resuspension or deposition of particles from occupants is the largest contributor of aerosolized particles in the NICU. In the current study, our petri dish ceiling analyses suggest a similar conclusion for settled particles but in this case based on biological data.

If occupancy is a key feature of the NICU environment, one would expect human-associated microbes to dominate in most room environments. We found that 5–10 OTUs account for most of the amplicon data and a majority of these are typically skin-, nose-, or fecal-associated (Fig. 2). The enrichment of human-associated taxa is likely due to tight control of the building envelope via HVAC treatment [37] combined with a strict cleaning schedule.

An interesting finding of this study related to the change in biomass and microbial community structure of the sink basins over the course of the week. We attribute this pattern to the room cleaning regime, which is more limited on weekend days than during the week. On Mondays, the sink biomass is highest (Fig. 3a) and communities are relatively uneven (Fig. 3b), presumably due to extensive growth of a few sink-associated taxa over the weekend. More intensive cleaning of the sink early in the week likely removes the majority of biomass, which is comprised of the sink-adapted taxa and enables detection of a wider diversity of low abundance, poorly adapted, or transient taxa.

The second question addressed in our study related to the taxa that dominate NICU surfaces. To investigate this, it was necessary to adapt a method to eliminate spurious contaminant-based signals in data from low biomass samples [29]. The ROTU cleaning method implemented here to clean data of spurious OTUs and contaminants in silico was made possible due to the availability of ddPCR quantification of negative controls. This capability is particularly important for NICU studies since the rooms are cleaned regularly, causing low biomass levels to be present in many samples. Some of the bacteria that we conclude were introduced in sample processing are skin-associated, although many types of taxa were encountered. After accounting for contamination, we conclude that human-associated taxa dominate most surfaces.

Human-associated taxa are likely sourced and trafficked throughout the NICU by healthcare providers [38], and many hand hygiene studies have reported as much [39]. Here, we implemented a machine learning classifier to address the possibility that infants and their caretakers shape the microbiome to be distinctive in each room. Our model reliably classified samples of unknown origin to their correct room-infant pair at an accuracy two times better than a recently published office microbiome study [25] and achieved predictive power five times better than random chance. This outcome suggests that NICU rooms are more personalized than other common built environments. There are typically a larger variety of activities and people in office spaces, and air treatment is less (lower air exchange rates and less filtration). The combination of less frequent cleaning, increased occupancy, and more unfiltered outdoor air supply drives many of the differences between other common indoor environments and the NICU. The more unique room signal based on NICU room microbes suggests a localized source of bacteria, since a more diffuse source would lower prediction accuracy. A similar result was recently described in a microbiome study conducted in a Chicago hospital [40]. Microbial community similarity increased between patients’ hand and floor samples over time, highlighting the exchange between patient and room. Interestingly, infants in this cohort are rarely removed from their isolettes, so room-specific microbiomes were likely mediated by healthcare providers, rather than direct infant interaction with surrounding room surfaces.

Finally, we tested for patterns of association between room occupants and NICU room environments. We found that many taxa driving our machine learning model for the room microbiome were from groups also present in the gut of the infant occupant. Other signals came from *Firmicutes* and *Actinobacteria* not affiliated with the infant gut and that were relatively uniquely detected in certain rooms. Focusing on the subset of taxa that are gut colonizers, we show a relatively high abundance of these taxa throughout the sampling campaign (Additional file 7). Episodes where persistent genera increase and 2–3 OTUs comprise > 30% of the data across all environments occurred several times throughout the study (e.g., in infants 9, 12, and 16). These OTUs are detected in low abundance in the room before detection in the gut (Fig. 5). Once in the infant gut, a far more favorable environment for growth and reproduction than on exposed hospital surfaces, bacterial density can reach nearly 10 billion cells per gram [5]. After a spike in relative abundance in the gut, we see these organisms increase in abundance in the room environment.

It is impossible to resolve room 16S rRNA amplicon data to the strain level in order to make claims that the same gut bloom resulted in a subsequent expanded appearance in the room. However, a recently published study from our group [12] conducted pooling of the room samples described here, performed metagenomic sequencing on sample pools, and showed room strains can share greater than 99.999% average nucleotide identity with infant gut strains. The *Klebsiella* OTU observed in this study, OTU_5, is likely the same strain isolated from infant 9’s room and gut, as room and gut genomes were nearly identical. Interestingly, similar types of *K. pneumoniae* found in the gut and room were detected years apart in different infants within this NICU [12].

## Conclusions

Based on the current study, we conclude that two factors shape room microbiomes. First, our taxa identifications and occupancy results extend prior findings of a strong link between human activity levels and room microbiology [10, 17, 18]. In fact, this connection appears to be strong enough to give rise to a relatively unique room microbiome character. Second, environmental stresses, likely associated with cleaning [10, 16, 41–43], likely selectively shape NICU microbiomes, primarily by selecting for microbial specialists that can both thrive in the gut and tolerate the NICU environment. While daily cleaning substantially lowers the bioburden in the NICU [44], the harshest cleaning methods cannot sterilize hospital surfaces [7]. Creative new approaches to displace or prevent entrenchment of these NICU specialists, possibly through prebiotic building materials or clever probiotics, may present opportunities to break the room occupant cycle.

## Additional files

Additional file 1: Biological and technical variation across ddPCR replicates. 16S rRNA gene template copy number was quantified via ddPCR for three petri dish dust collectors suspended from the drop ceiling in each infant’s room. Each dot reflects the average across triplicate runs. Each infant set is labeled at the top of the plot facets. (PNG 152 kb)

Additional file 2: Biomass in air samples from a NICU. 16S rRNA gene template copy number was quantified via ddPCR. Each dot reflects the average across triplicate runs. Gray diamonds represent averages per environment. Bioaerosol measurements in (A) are separated by small and large size fractions (particles 1–4 and > 4 μm, respectively). HVAC samples in (B) were collected from the exterior facet of the HVAC system and represent pretreated air. Counts are normalized per sample per day of collection. (PDF 16 kb)

Additional file 3: SourceTracker reveals human skin is dominant source of NICU microbes. American gut skin, oral, and fecal samples were used as “sources,” and NICU room samples were used as “sinks” and input into the SourceTracker software. Plotted on the *y*-axis is the mean relative contribution of each human-associated source to each environmental sample. (PDF 7 kb)

Additional file 4: Alpha diversity in a NICU. 16S rRNA gene amplicon data was used to calculate number of OTUs per environment (a–c) and the Shannon diversity (d–f). (PDF 10 kb)

Additional file 5: Top 10 most important taxa driving the machine learning model. The top 10 most important variables driving the SVM model are plotted for each infant. On the *y*-axis, “Abundance,” notes the relative importance. (PDF 202 kb)

Additional file 6: Fecal sample community composition. Plotted in each panel is the community composition of each infant’s fecal samples derived from metagenomics data. (PDF 114 kb)

Additional file 7: Episodic increases in persistent taxa. The “average” panel represents 16S rRNA gene amplicon data averaged across all samples at each time point per infant. The “armrest” and “sink_basins” panel is the same data but without averaging across environments. The red line highlights the time point in which an increase of *Enterobacteriaceae* was detected in infant 9’s gut. Samples are plotted in chronological order on the *x*-axis. (PDF 433 kb)
