# Supplementary figures and images for: The developing premature infant gut microbiome is a major factor shaping the microbiome of neonatal intensive care unit rooms
Source: Microbiome. 2018 Jun 20;6:112. doi: 10.1186/s40168-018-0493-5 (PMC6011520; doi:10.1186/s40168-018-0493-5)

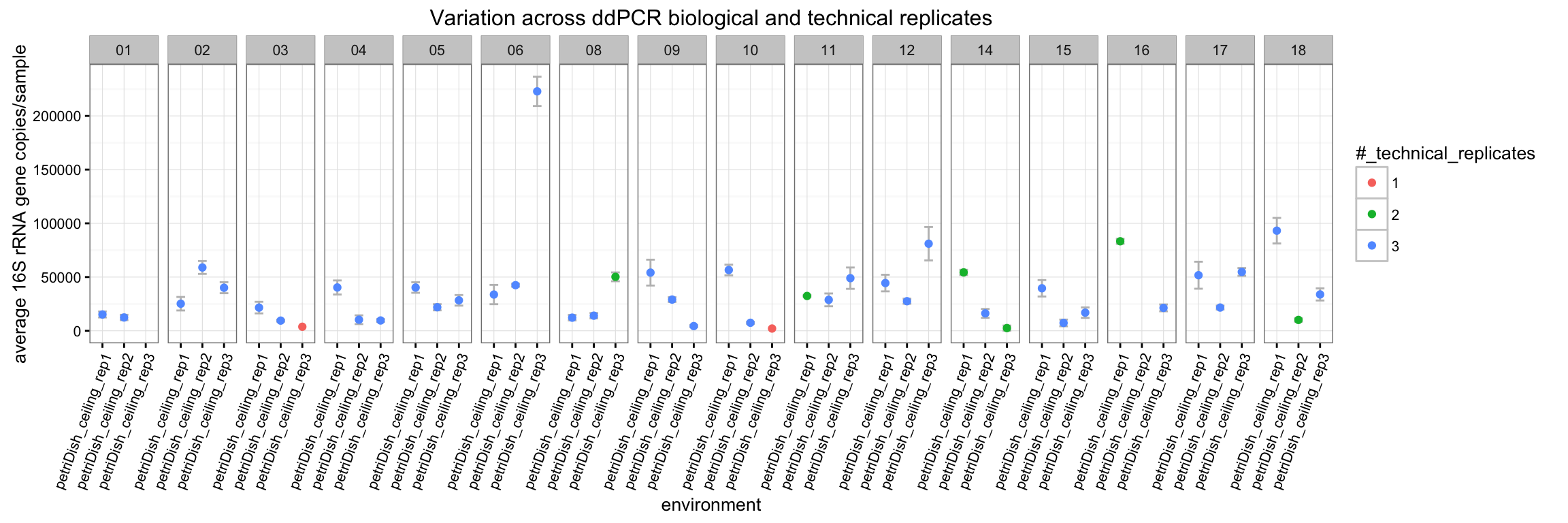

Supplement: Supplementary file 1 — Biological and technical variation across ddPCR replicates. 16S rRNA gene template copy number was quantified via ddPCR for three petri dish dust collectors suspended from the drop ceiling in each infant’s room. Each dot reflects the average across triplicate runs. Each infant set is labeled at the top of the plot facets. (PNG 152 kb) [file 40168_2018_493_MOESM1_ESM.png]

**A**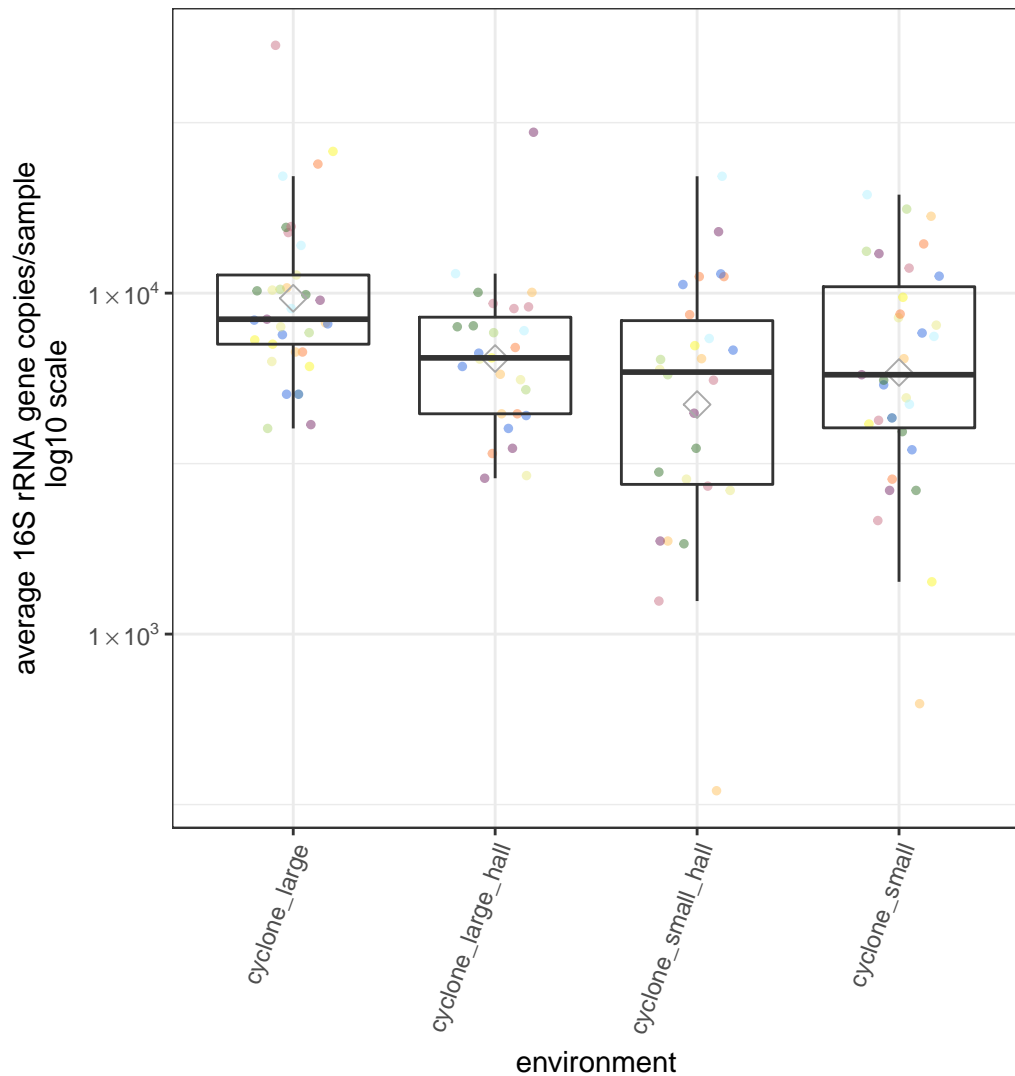**B**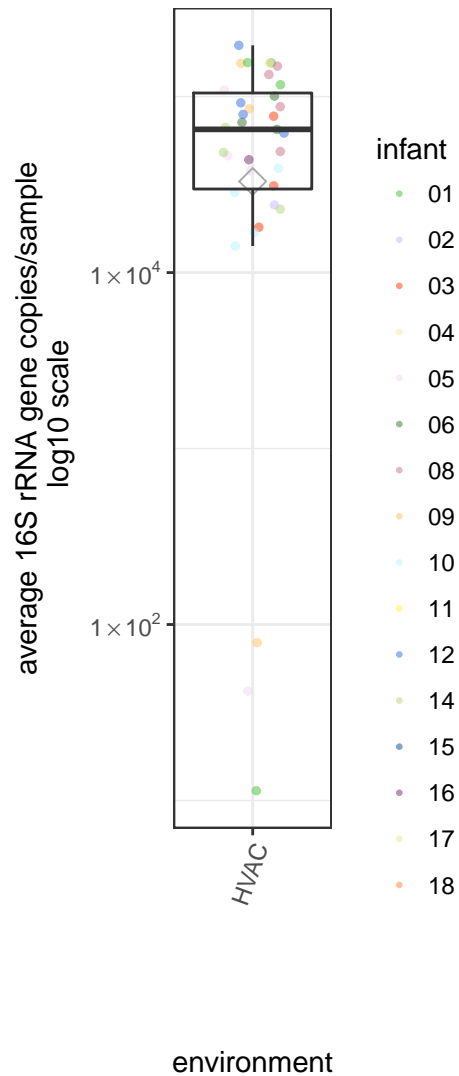

Supplement: Supplementary file 2 — Biomass in air samples from a NICU. 16S rRNA gene template copy number was quantified via ddPCR. Each dot reflects the average across triplicate runs. Gray diamonds represent averages per environment. Bioaerosol measurements in (A) are separated by small and large size fractions (particles 1–4 and > 4 μm, respectively). HVAC samples in (B) were collected from the exterior facet of the HVAC system and represent pretreated air. Counts are normalized per sample per day of collection. (PDF 16 kb) [file 40168_2018_493_MOESM2_ESM.pdf]

relative source contribution

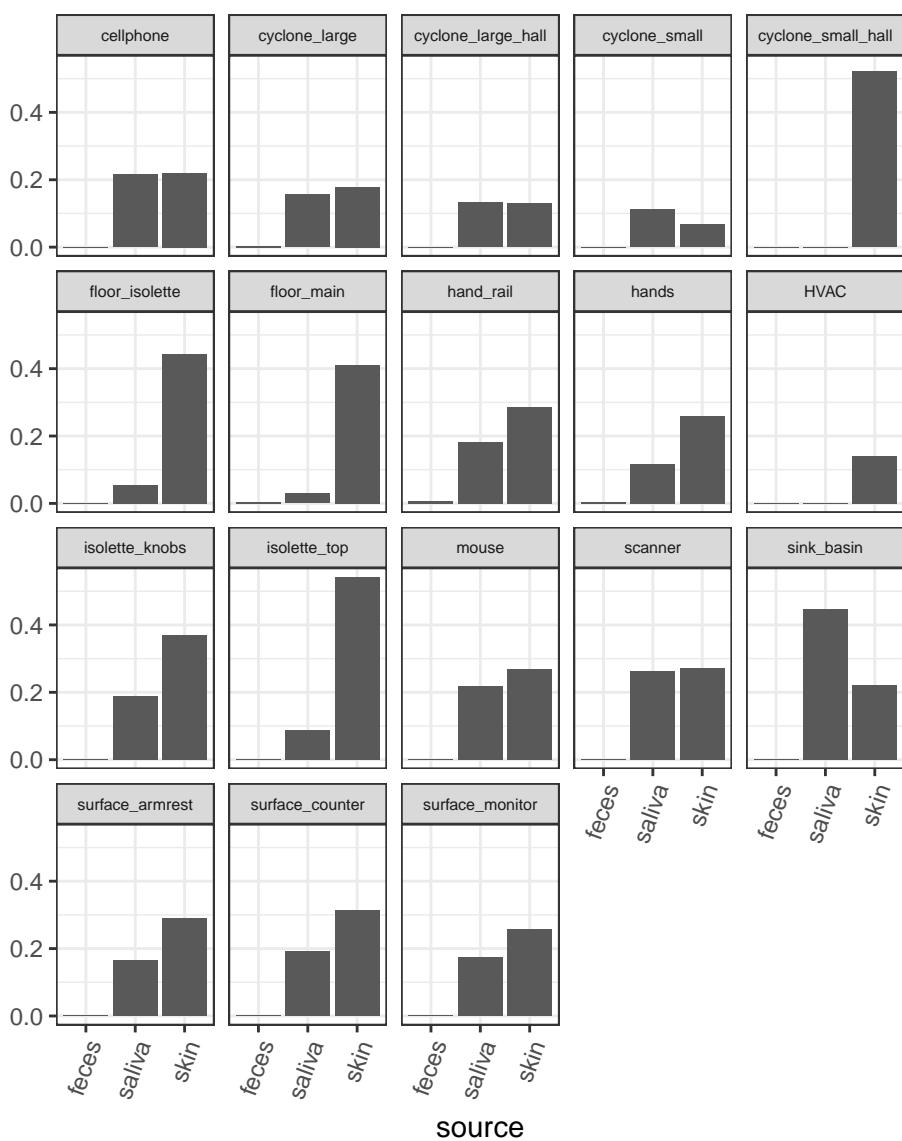

Supplement: Supplementary file 3 — SourceTracker reveals human skin is dominant source of NICU microbes. American gut skin, oral, and fecal samples were used as “sources,” and NICU room samples were used as “sinks” and input into the SourceTracker software. Plotted on the y-axis is the mean relative contribution of each human-associated source to each environmental sample. (PDF 7 kb) [file 40168_2018_493_MOESM3_ESM.pdf]

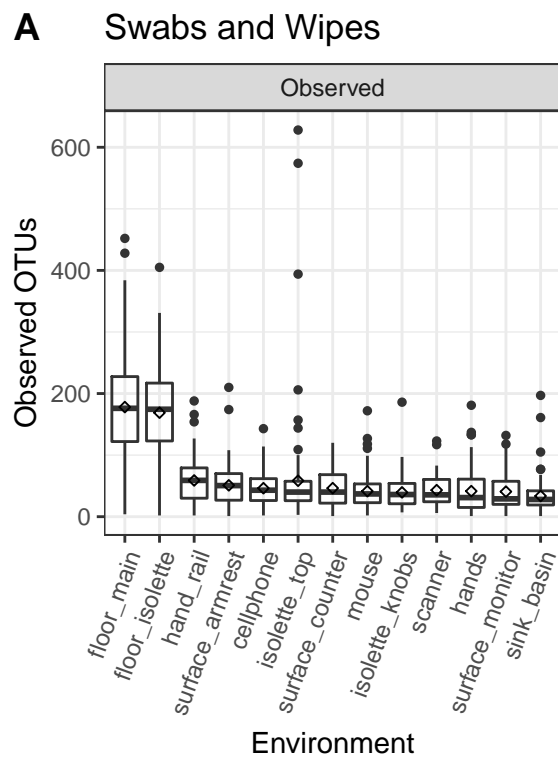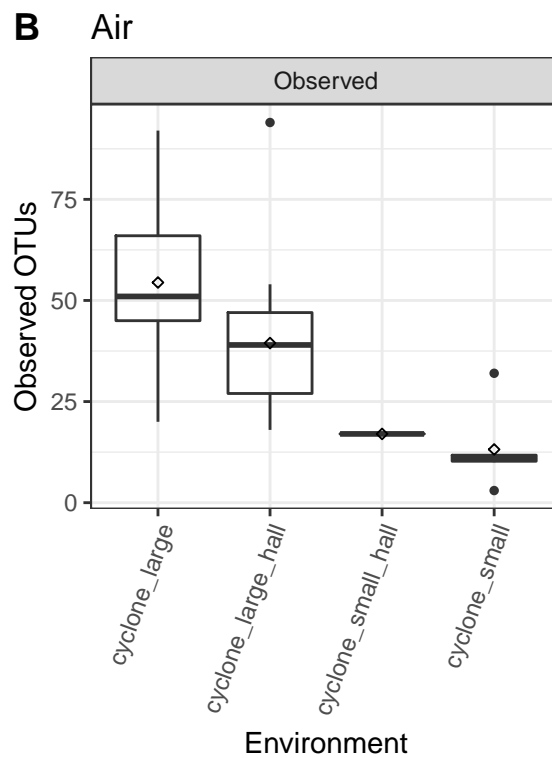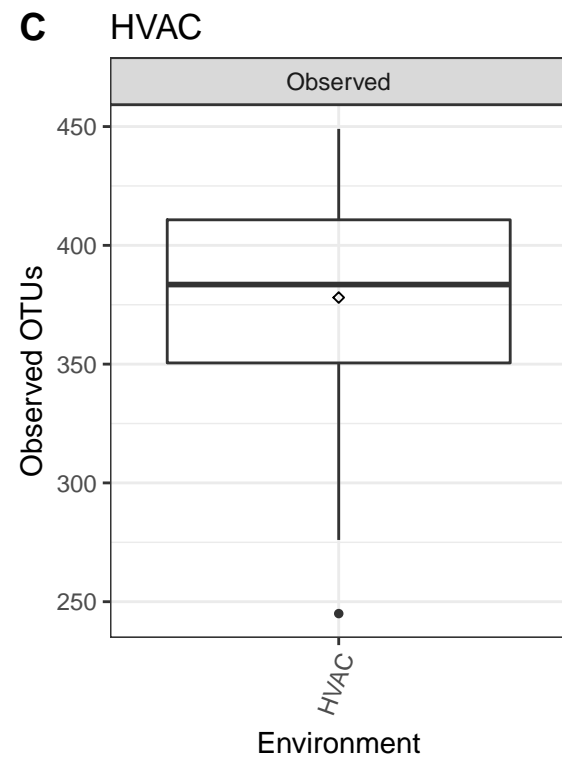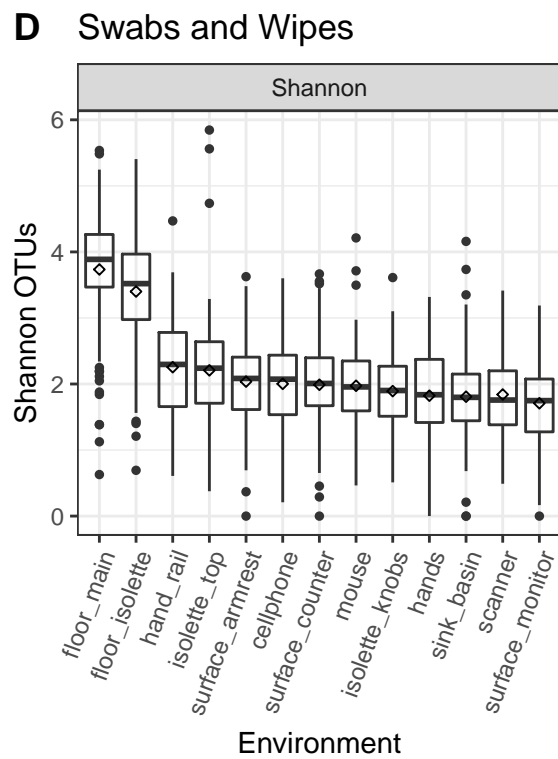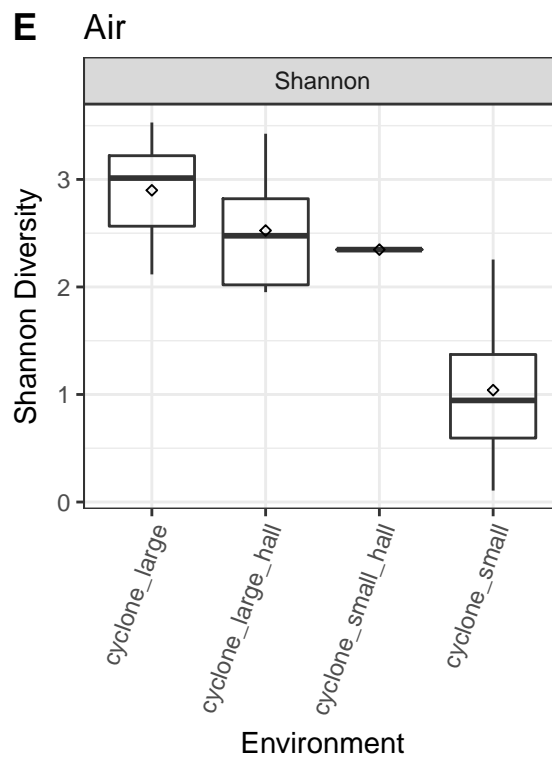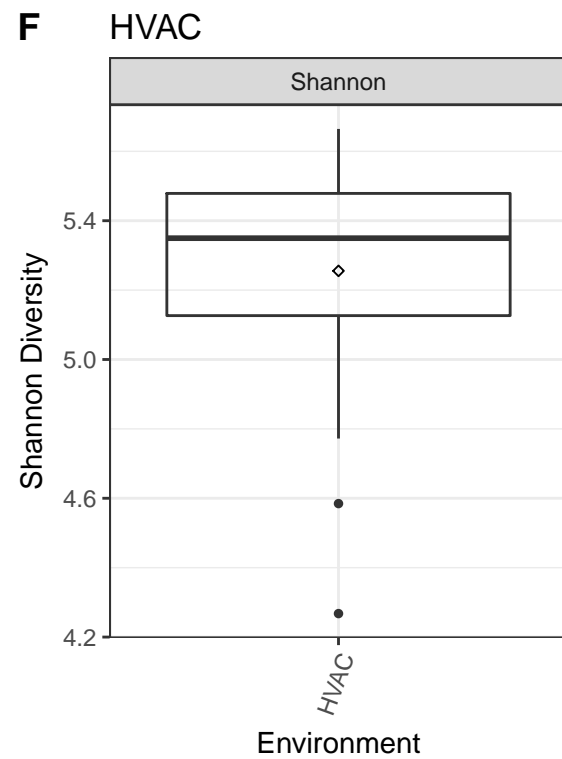

Supplement: Supplementary file 4 — Alpha diversity in a NICU. 16S rRNA gene amplicon data was used to calculate number of OTUs per environment (a–c) and the Shannon diversity (d–f). (PDF 10 kb) [file 40168_2018_493_MOESM4_ESM.pdf]

Top 10 most influential OTUs from SVM

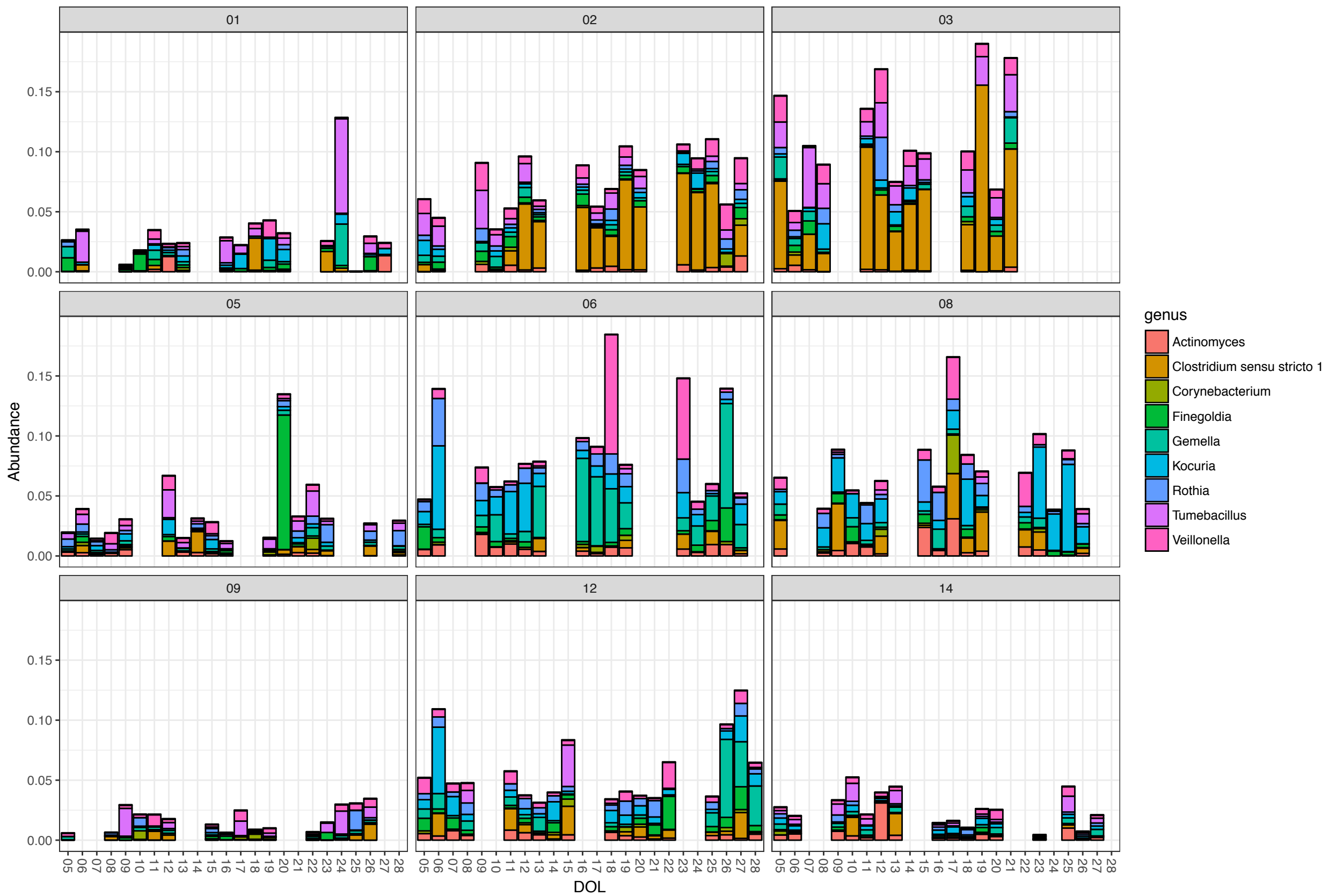

Supplement: Supplementary file 5 — Top 10 most important taxa driving the machine learning model. The top 10 most important variables driving the SVM model are plotted for each infant. On the y-axis, “Abundance,” notes the relative importance. (PDF 202 kb) [file 40168_2018_493_MOESM5_ESM.pdf]

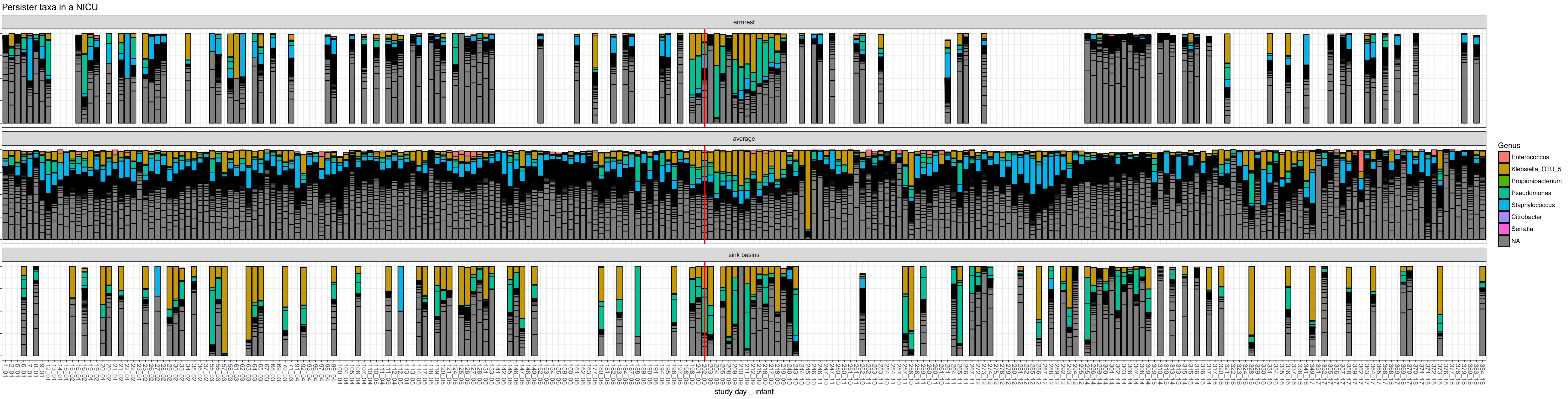

Supplement: Supplementary file 7 — Episodic increases in persistent taxa. The “average” panel represents 16S rRNA gene amplicon data averaged across all samples at each time point per infant. The “armrest” and “sink_basins” panel is the same data but without averaging across environments. The red line highlights the time point in which an increase of Enterobacteriaceae was detected in infant 9’s gut. Samples are plotted in chronological order on the x-axis. (PDF 433 kb) [file 40168_2018_493_MOESM7_ESM.pdf]
