# Supplementary material for: The developing premature infant gut microbiome is a major factor shaping the microbiome of neonatal intensive care unit rooms
Source: Microbiome. 2018 Jun 20;6:112. doi: 10.1186/s40168-018-0493-5 (PMC6011520; doi:10.1186/s40168-018-0493-5)
Supplement: Supplementary file 6 — Fecal sample community composition. Plotted in each panel is the community composition of each infant’s fecal samples derived from metagenomics data. (PDF 114 kb) [file 40168_2018_493_MOESM6_ESM.pdf]

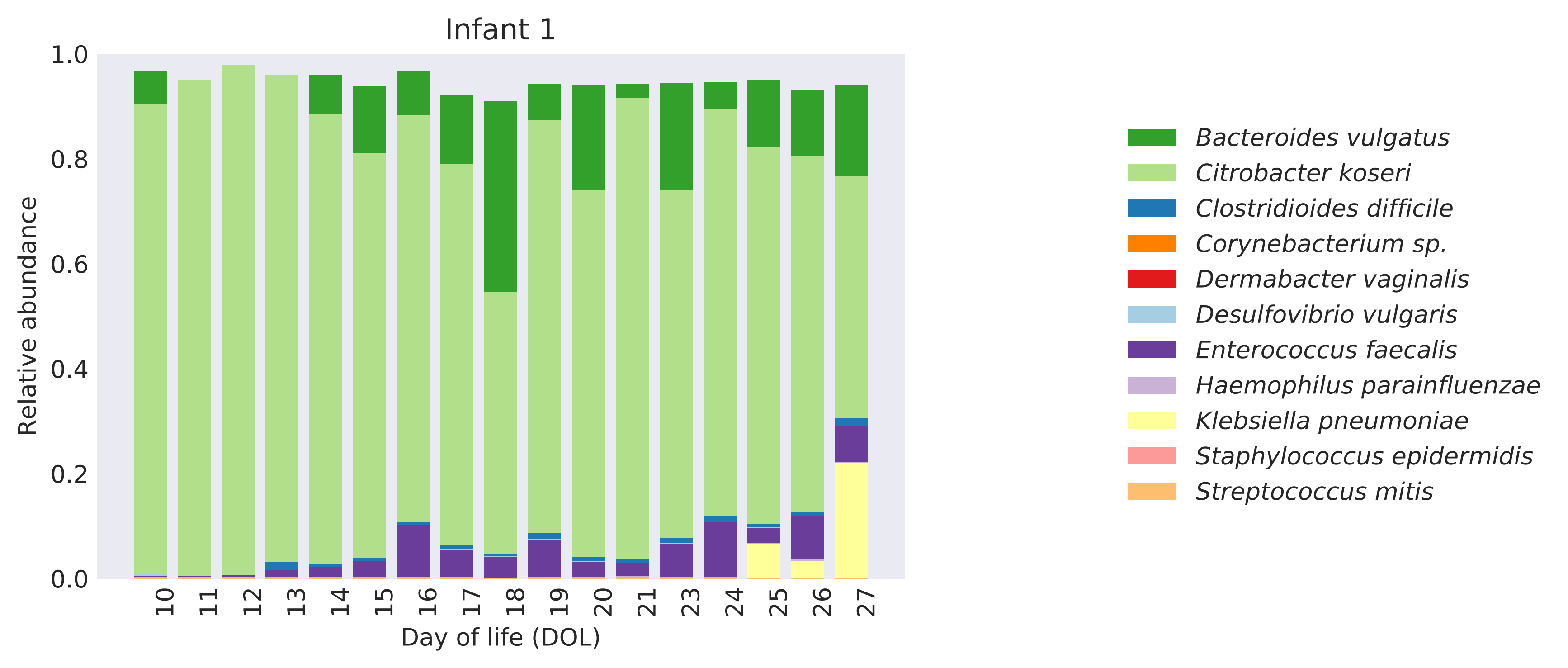

Infant 1
Relative abundance
1.0
0.8
0.6
0.4
0.2
0.0
10
11
12
13
14
15
16
17
18
19
20
21
23
24
25
26
27
Day of life (DOL)
Bacteroides vulgatus
Citrobacter koseri
Clostridioides difficile
Corynebacterium sp.
Dermabacter vaginalis
Desulfovibrio vulgaris
Enterococcus faecalis
Haemophilus parainfluenzae
Klebsiella pneumoniae
Staphylococcus epidermidis
Streptococcus mitis

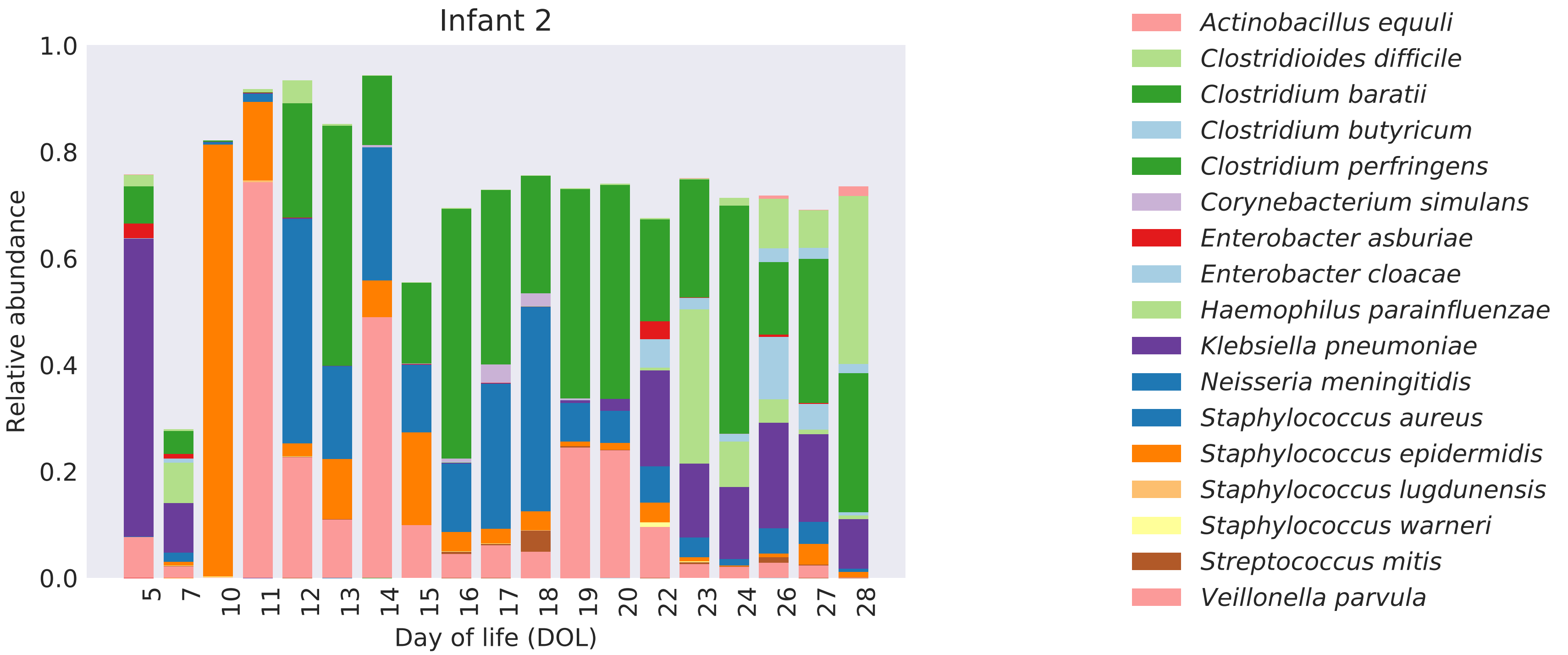

Infant 2
Relative abundance
1.0
0.8
0.6
0.4
0.2
0.0
5
7
10
11
12
13
14
15
16
17
18
19
20
22
23
24
26
27
28
Day of life (DOL)
Actinobacillus equuli
Clostridioides difficile
Clostridium baratii
Clostridium butyricum
Clostridium perfringens
Corynebacterium simulans
Enterobacter asburiae
Enterobacter cloacae
Haemophilus parainfluenzae
Klebsiella pneumoniae
Neisseria meningitidis
Staphylococcus aureus
Staphylococcus epidermidis
Staphylococcus lugdunensis
Staphylococcus warneri
Streptococcus mitis
Veillonella parvula

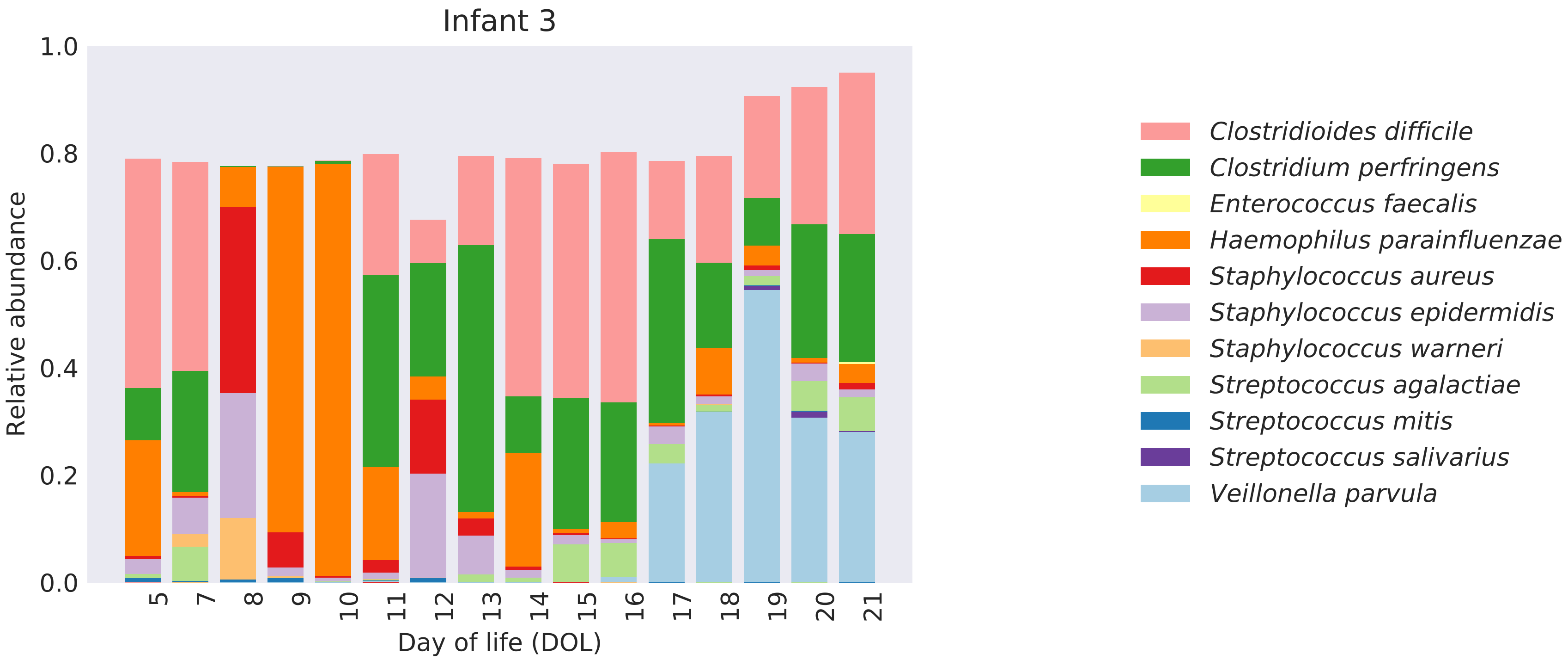

Infant 3
Relative abundance
Day of life (DOL)
1.0
0.8
0.6
0.4
0.2
0.0
5
7
8
9
10
11
12
13
14
15
16
17
18
19
20
21
Clostridioides difficile
Clostridium perfringens
Enterococcus faecalis
Haemophilus parainfluenzae
Staphylococcus aureus
Staphylococcus epidermidis
Staphylococcus warneri
Streptococcus agalactiae
Streptococcus mitis
Streptococcus salivarius
Veillonella parvula

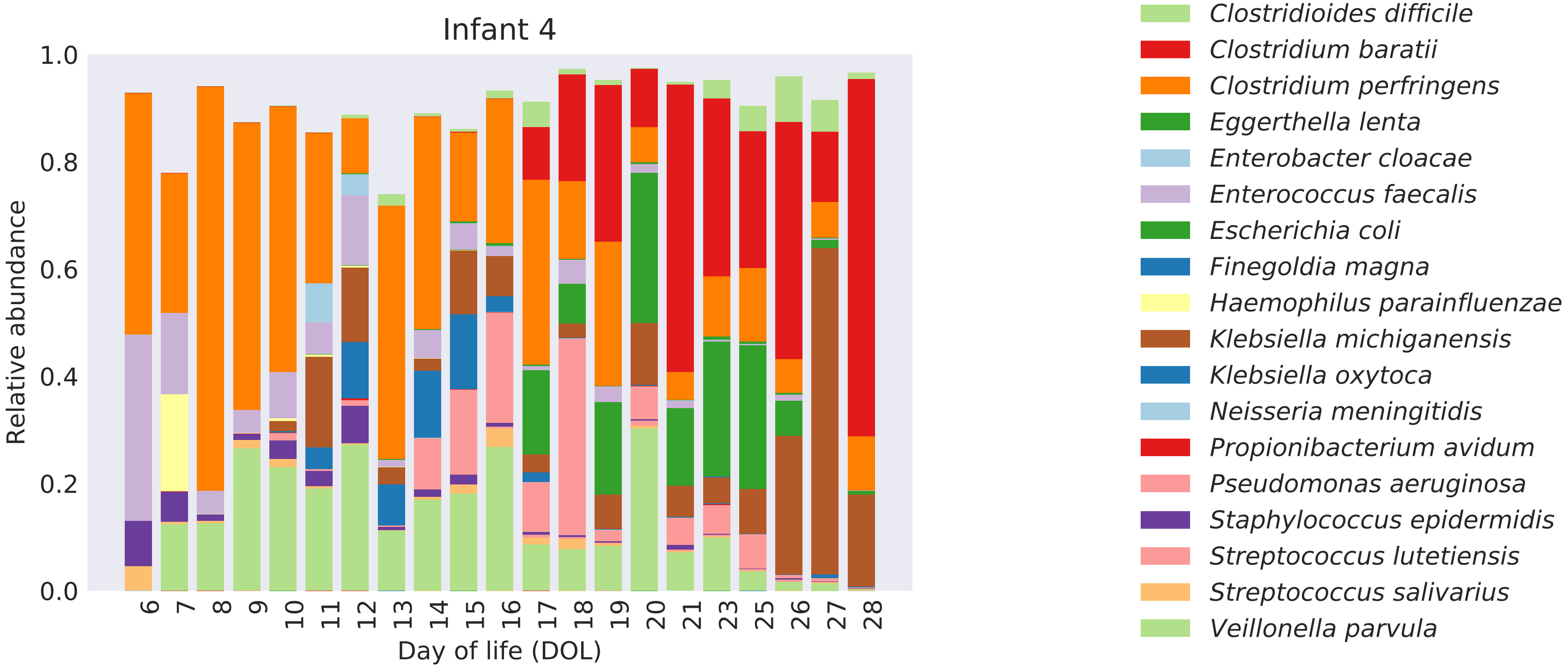
Infant 4
Relative abundance
1.0
0.8
0.6
0.4
0.2
0.0
6
7
8
9
10
11
12
13
14
15
16
17
18
19
20
21
23
25
26
27
28
Day of life (DOL)
Clostridioides difficile
Clostridium baratii
Clostridium perfringens
Eggerthella lenta
Enterobacter cloacae
Enterococcus faecalis
Escherichia coli
Finegoldia magna
Haemophilus parainfluenzae
Klebsiella michiganensis
Klebsiella oxytoca
Neisseria meningitidis
Propionibacterium avidum
Pseudomonas aeruginosa
Staphylococcus epidermidis
Streptococcus lutetiensis
Streptococcus salivarius
Veillonella parvula

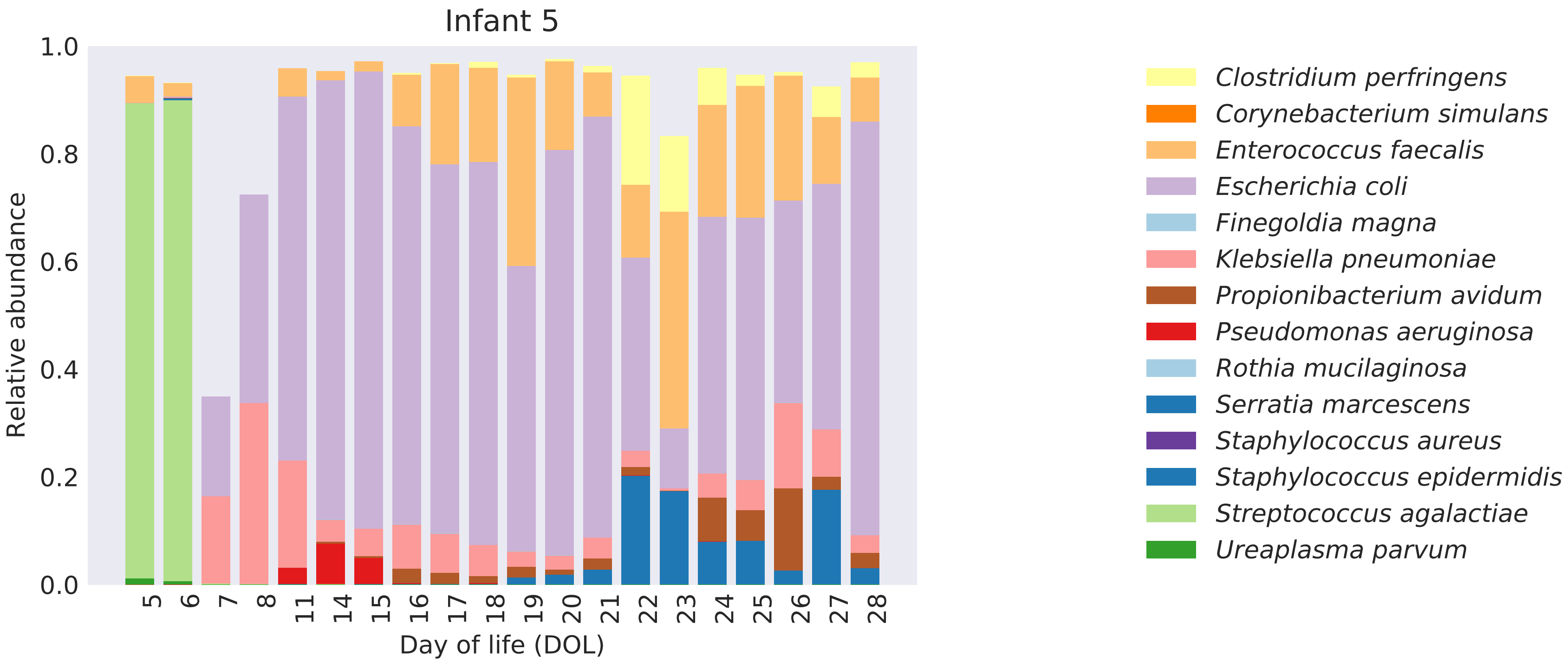

Infant 5
Relative abundance
1.0
0.8
0.6
0.4
0.2
0.0
5
6
7
8
11
14
15
16
17
18
19
20
21
22
23
24
25
26
27
28
Day of life (DOL)
Clostridium perfringens
Corynebacterium simulans
Enterococcus faecalis
Escherichia coli
Finegoldia magna
Klebsiella pneumoniae
Propionibacterium avidum
Pseudomonas aeruginosa
Rothia mucilaginosa
Serratia marcescens
Staphylococcus aureus
Staphylococcus epidermidis
Streptococcus agalactiae
Ureaplasma parvum

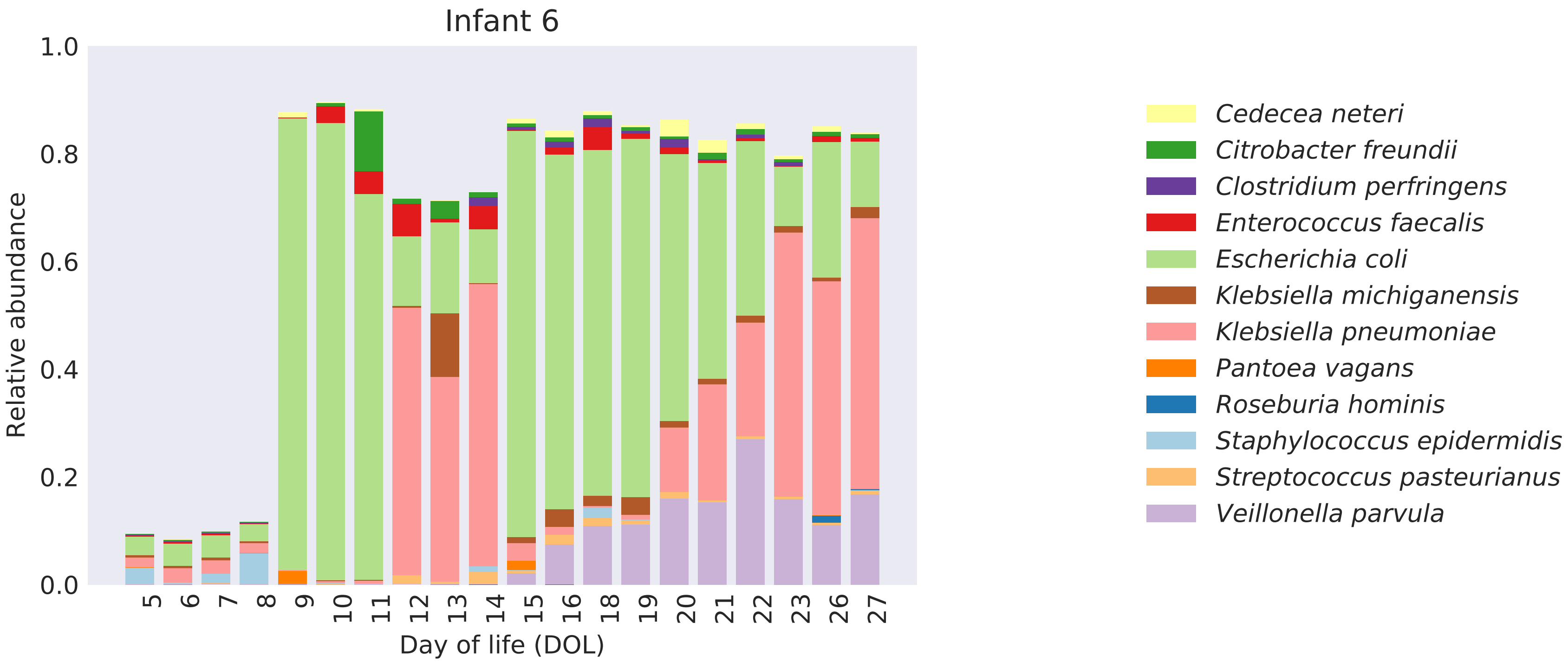

Infant 6
Relative abundance
1.0
0.8
0.6
0.4
0.2
0.0
5
6
7
8
9
10
11
12
13
14
15
16
18
19
20
21
22
23
26
27
Day of life (DOL)
Cedecea neteri
Citrobacter freundii
Clostridium perfringens
Enterococcus faecalis
Escherichia coli
Klebsiella michiganensis
Klebsiella pneumoniae
Pantoea vagans
Roseburia hominis
Staphylococcus epidermidis
Streptococcus pasteurianus
Veillonella parvula

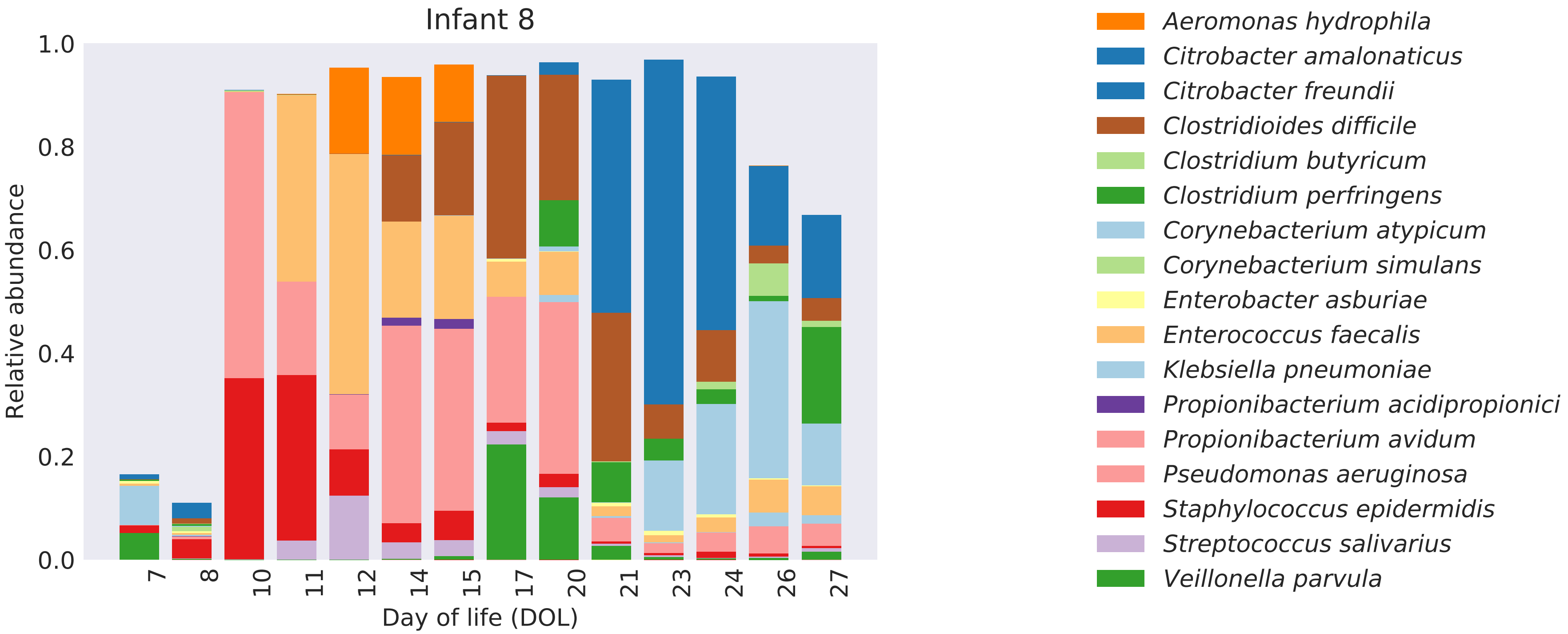
Infant 8
Relative abundance
1.0
0.8
0.6
0.4
0.2
0.0
7
8
10
11
12
14
15
17
20
21
23
24
26
27
Day of life (DOL)
Aeromonas hydrophila
Citrobacter amalonaticus
Citrobacter freundii
Clostridioides difficile
Clostridium butyricum
Clostridium perfringens
Corynebacterium atypicum
Corynebacterium simulans
Enterobacter asburiae
Enterococcus faecalis
Klebsiella pneumoniae
Propionibacterium acidipropionici
Propionibacterium avidum
Pseudomonas aeruginosa
Staphylococcus epidermidis
Streptococcus salivarius
Veillonella parvula

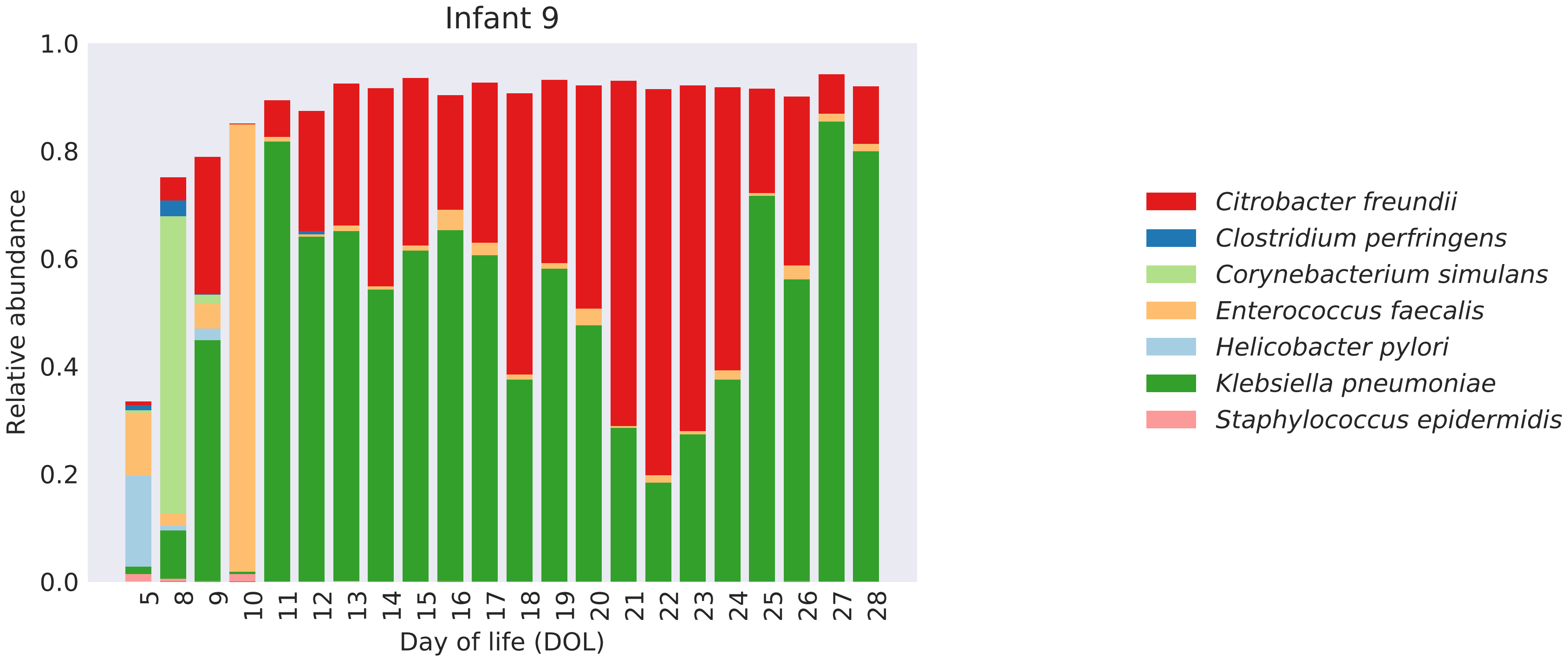

Infant 9
Relative abundance
1.0
0.8
0.6
0.4
0.2
0.0
5
8
9
10
11
12
13
14
15
16
17
18
19
20
21
22
23
24
25
26
27
28
Day of life (DOL)
Citrobacter freundii
Clostridium perfringens
Corynebacterium simulans
Enterococcus faecalis
Helicobacter pylori
Klebsiella pneumoniae
Staphylococcus epidermidis

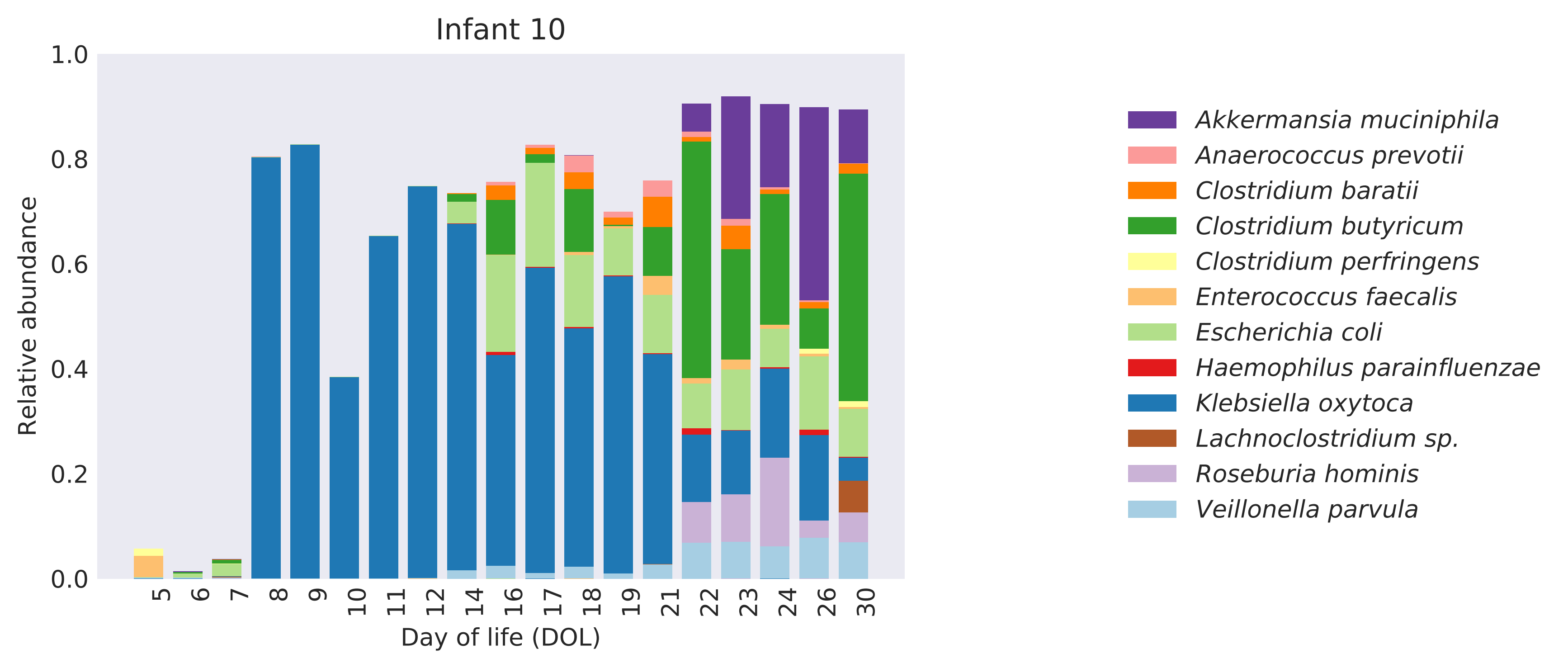
Infant 10
Relative abundance
1.0
0.8
0.6
0.4
0.2
0.0
5
6
7
8
9
10
11
12
14
16
17
18
19
21
22
23
24
26
30
Day of life (DOL)
Akkermansia muciniphila
Anaerococcus prevotii
Clostridium baratii
Clostridium butyricum
Clostridium perfringens
Enterococcus faecalis
Escherichia coli
Haemophilus parainfluenzae
Klebsiella oxytoca
Lachnoclostridium sp.
Roseburia hominis
Veillonella parvula

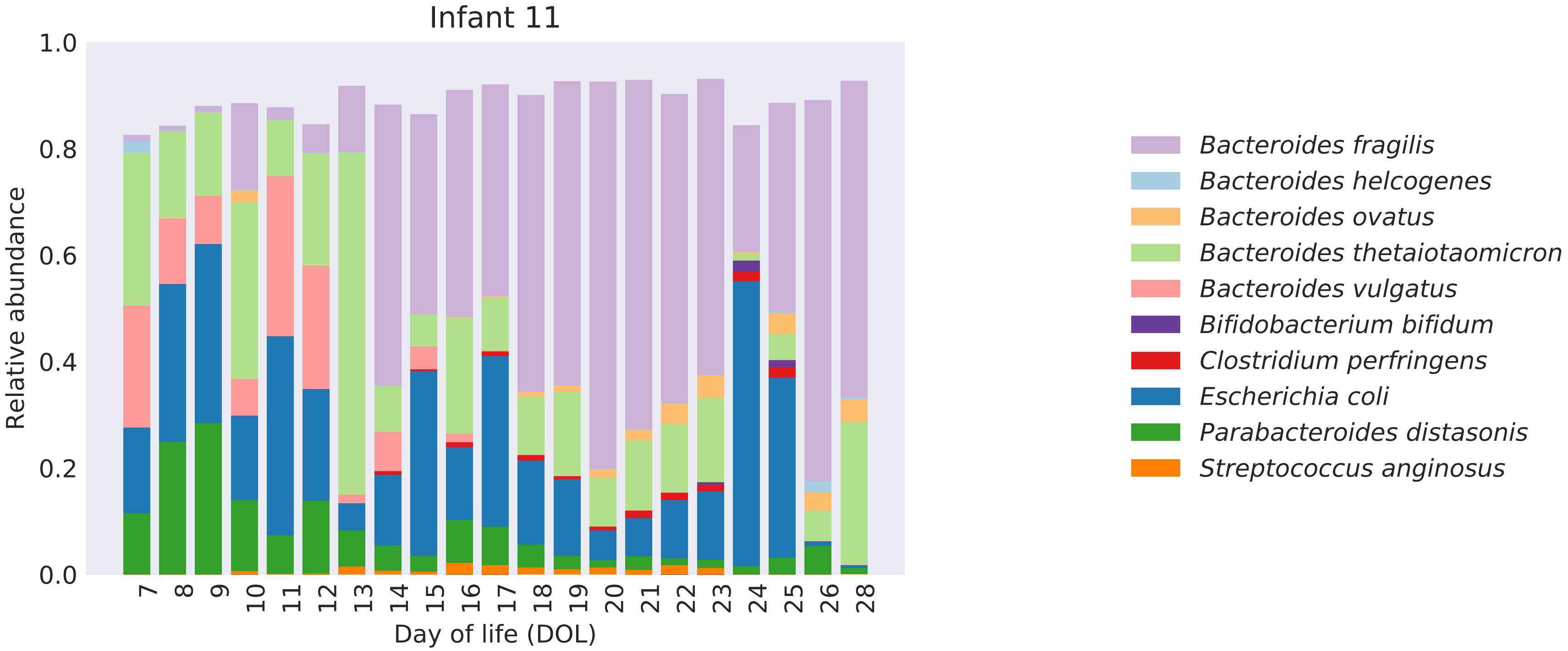

Infant 11
Relative abundance
1.0
0.8
0.6
0.4
0.2
0.0
7
8
9
10
11
12
13
14
15
16
17
18
19
20
21
22
23
24
25
26
28
Day of life (DOL)
Bacteroides fragilis
Bacteroides helcogenes
Bacteroides ovatus
Bacteroides thetaiotaomicron
Bacteroides vulgatus
Bifidobacterium bifidum
Clostridium perfringens
Escherichia coli
Parabacteroides distasonis
Streptococcus anginosus

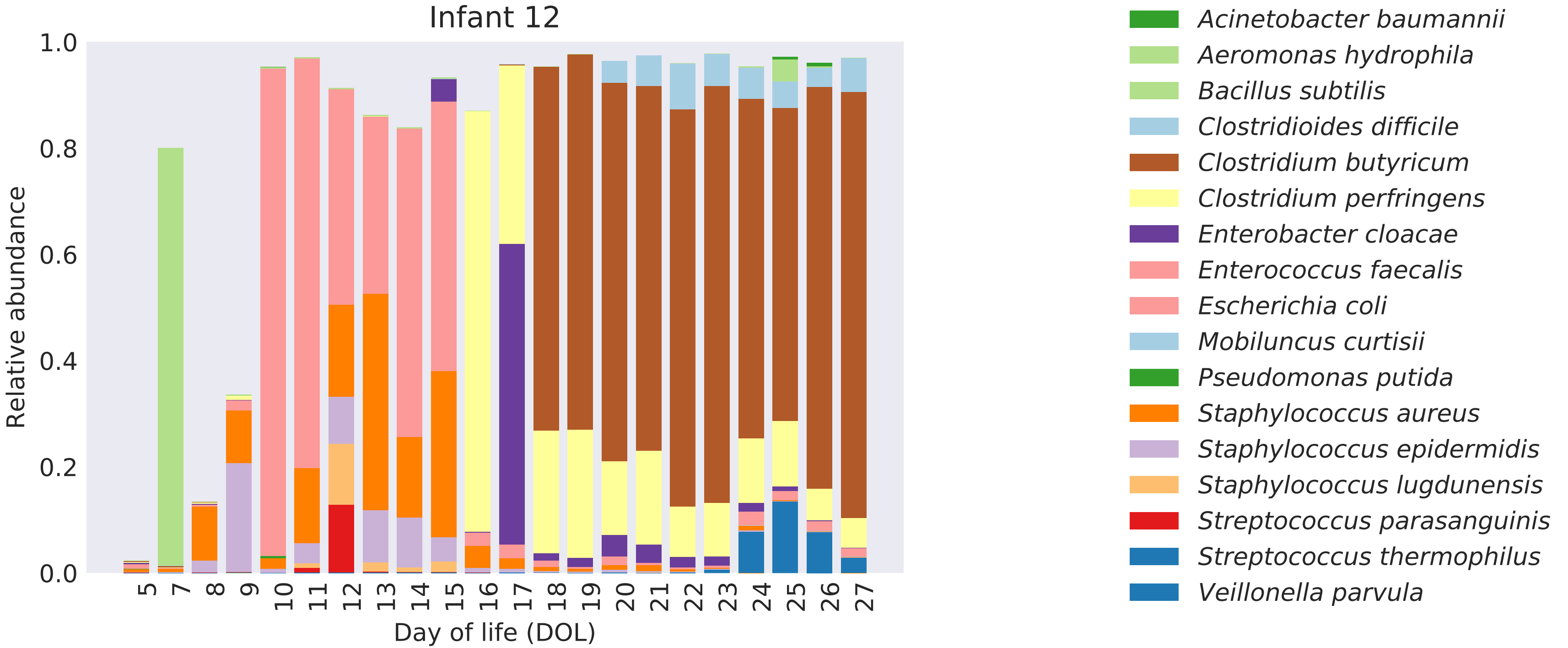

Infant 12
Relative abundance
1.0
0.8
0.6
0.4
0.2
0.0
5
7
8
9
10
11
12
13
14
15
16
17
18
19
20
21
22
23
24
25
26
27
Day of life (DOL)
Acinetobacter baumannii
Aeromonas hydrophila
Bacillus subtilis
Clostridioides difficile
Clostridium butyricum
Clostridium perfringens
Enterobacter cloacae
Enterococcus faecalis
Escherichia coli
Mobiluncus curtisii
Pseudomonas putida
Staphylococcus aureus
Staphylococcus epidermidis
Staphylococcus lugdunensis
Streptococcus parasanguinis
Streptococcus thermophilus
Veillonella parvula

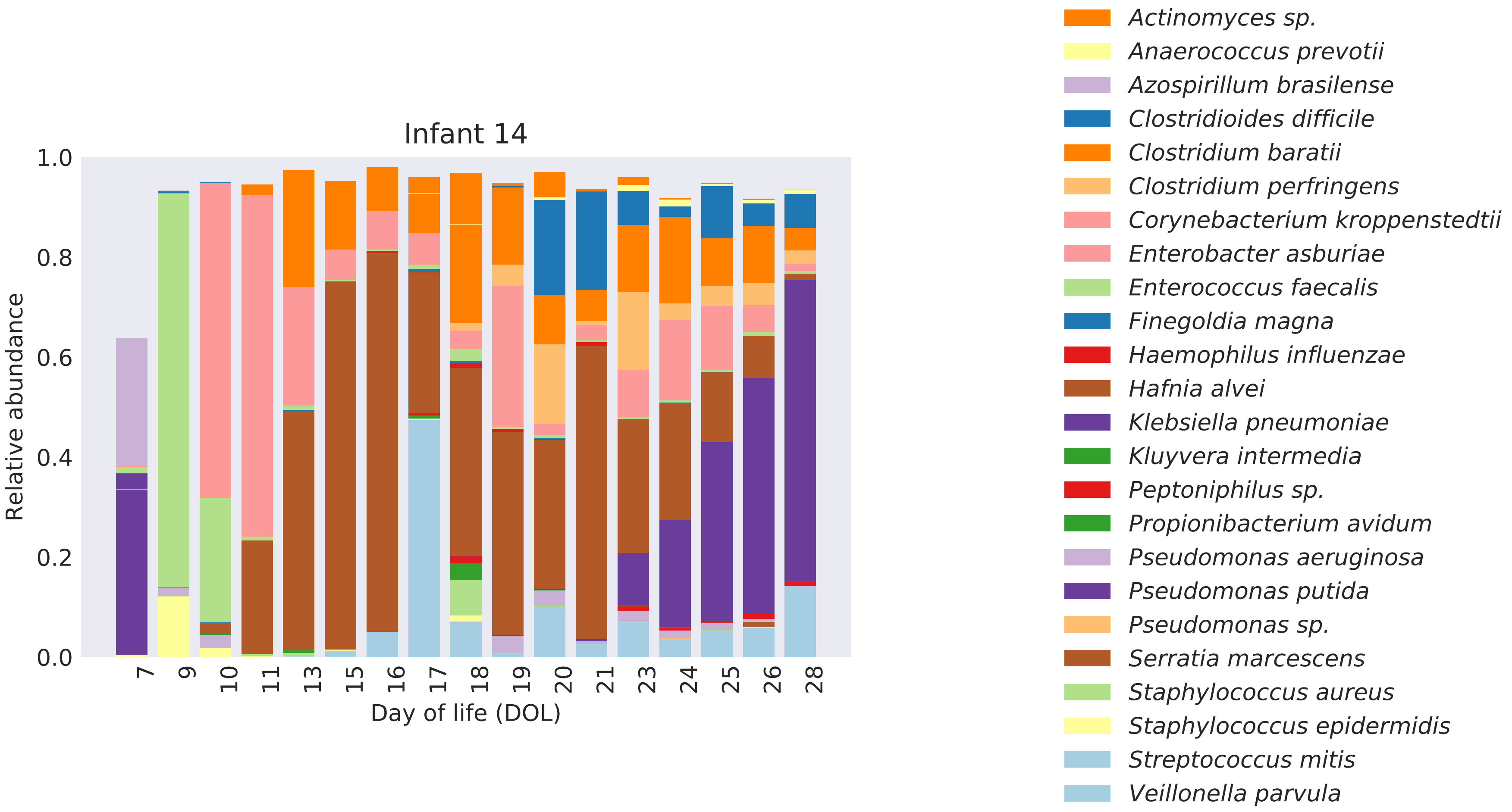

Infant 14
Relative abundance
1.0
0.8
0.6
0.4
0.2
0.0
7
9
10
11
13
15
16
17
18
19
20
21
23
24
25
26
28
Day of life (DOL)
Actinomyces sp.
Anaerococcus prevotii
Azospirillum brasilense
Clostridioides difficile
Clostridium baratii
Clostridium perfringens
Corynebacterium kroppenstedtii
Enterobacter asburiae
Enterococcus faecalis
Finegoldia magna
Haemophilus influenzae
Hafnia alvei
Klebsiella pneumoniae
Kluyvera intermedia
Peptoniphilus sp.
Propionibacterium avidum
Pseudomonas aeruginosa
Pseudomonas putida
Pseudomonas sp.
Serratia marcescens
Staphylococcus aureus
Staphylococcus epidermidis
Streptococcus mitis
Veillonella parvula

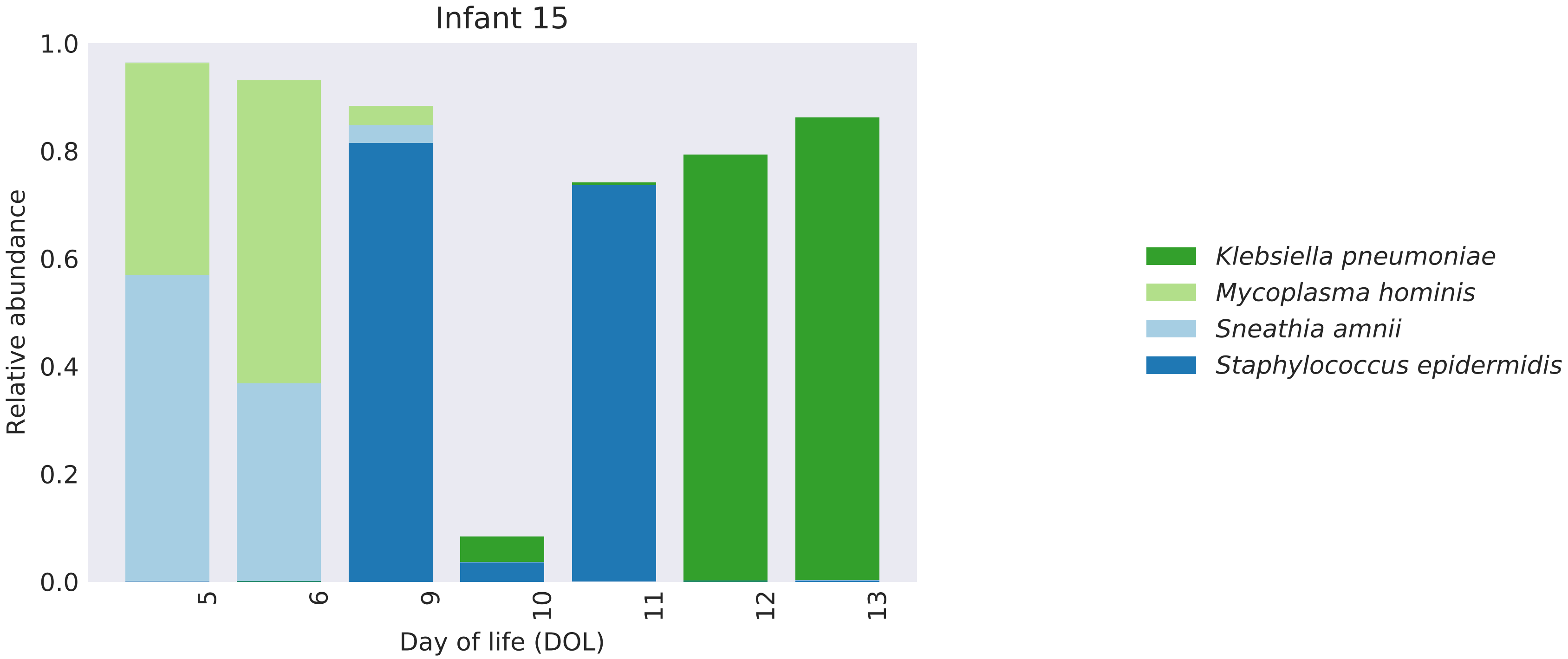
Infant 15
1.0
0.8
0.6
0.4
0.2
0.0
Relative abundance
5
6
9
10
11
12
13
Day of life (DOL)
Klebsiella pneumoniae
Mycoplasma hominis
Sneathia amnii
Staphylococcus epidermidis

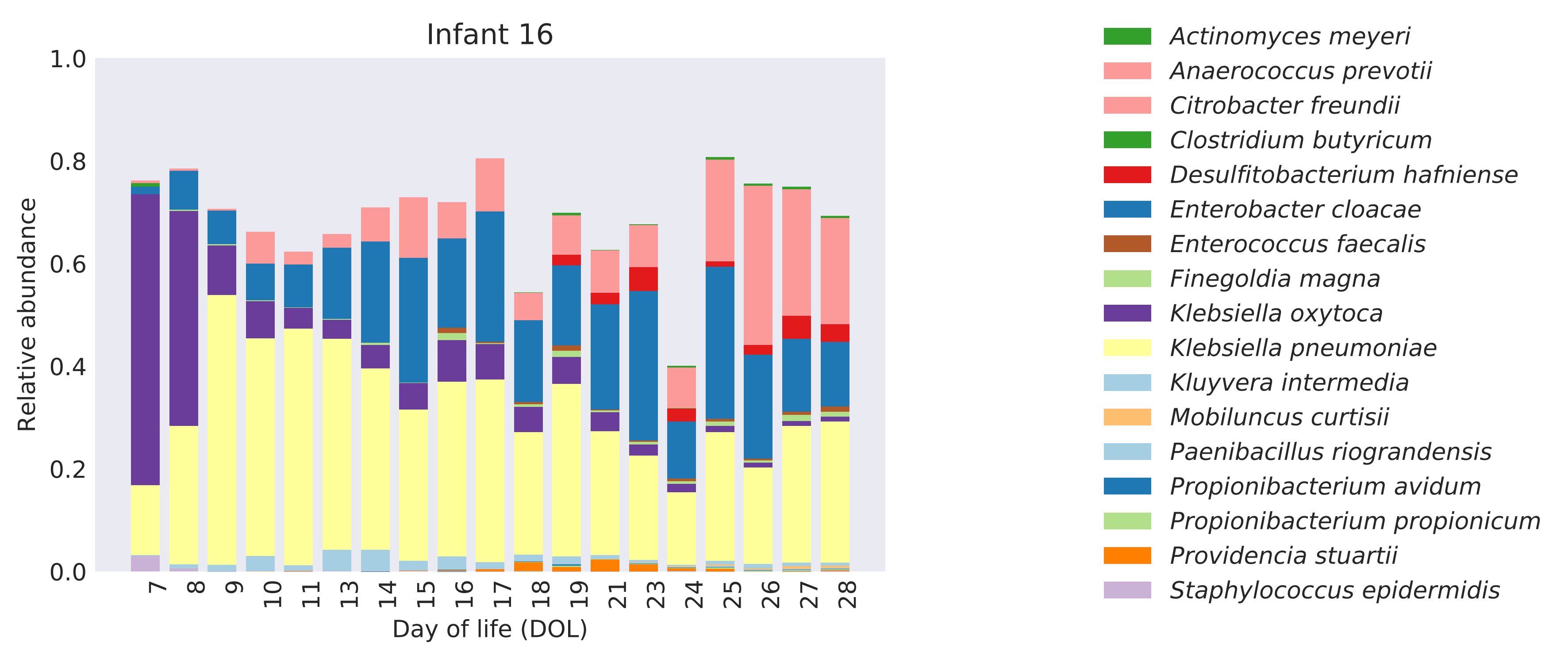

Infant 16
Relative abundance
1.0
0.8
0.6
0.4
0.2
0.0
7
8
9
10
11
13
14
15
16
17
18
19
21
23
24
25
26
27
28
Day of life (DOL)
Actinomyces meyeri
Anaerococcus prevotii
Citrobacter freundii
Clostridium butyricum
Desulfitobacterium hafniense
Enterobacter cloacae
Enterococcus faecalis
Finegoldia magna
Klebsiella oxytoca
Klebsiella pneumoniae
Kluyvera intermedia
Mobiluncus curtisii
Paenibacillus riograndensis
Propionibacterium avidum
Propionibacterium propionicum
Providencia stuartii
Staphylococcus epidermidis

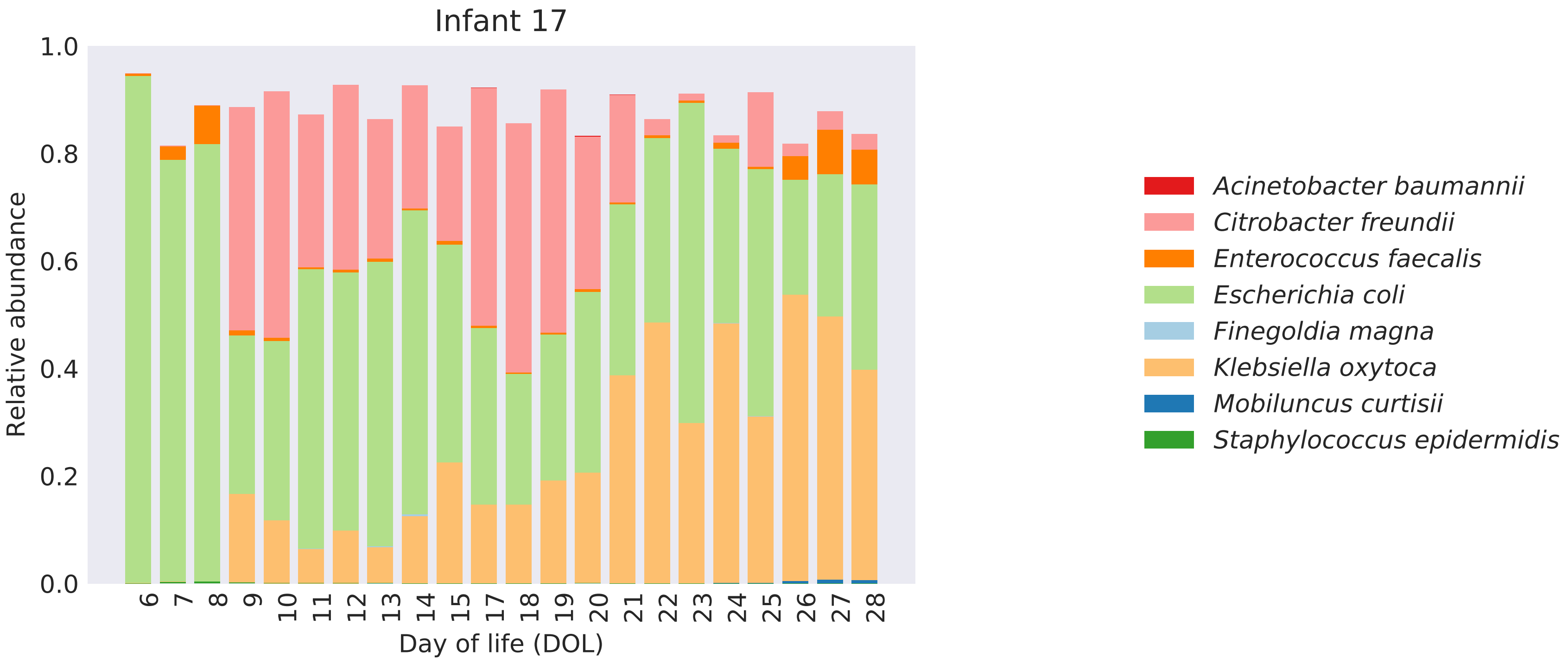

Infant 17
Relative abundance
1.0
0.8
0.6
0.4
0.2
0.0
6
7
8
9
10
11
12
13
14
15
17
18
19
20
21
22
23
24
25
26
27
28
Day of life (DOL)
Acinetobacter baumannii
Citrobacter freundii
Enterococcus faecalis
Escherichia coli
Finegoldia magna
Klebsiella oxytoca
Mobiluncus curtisii
Staphylococcus epidermidis

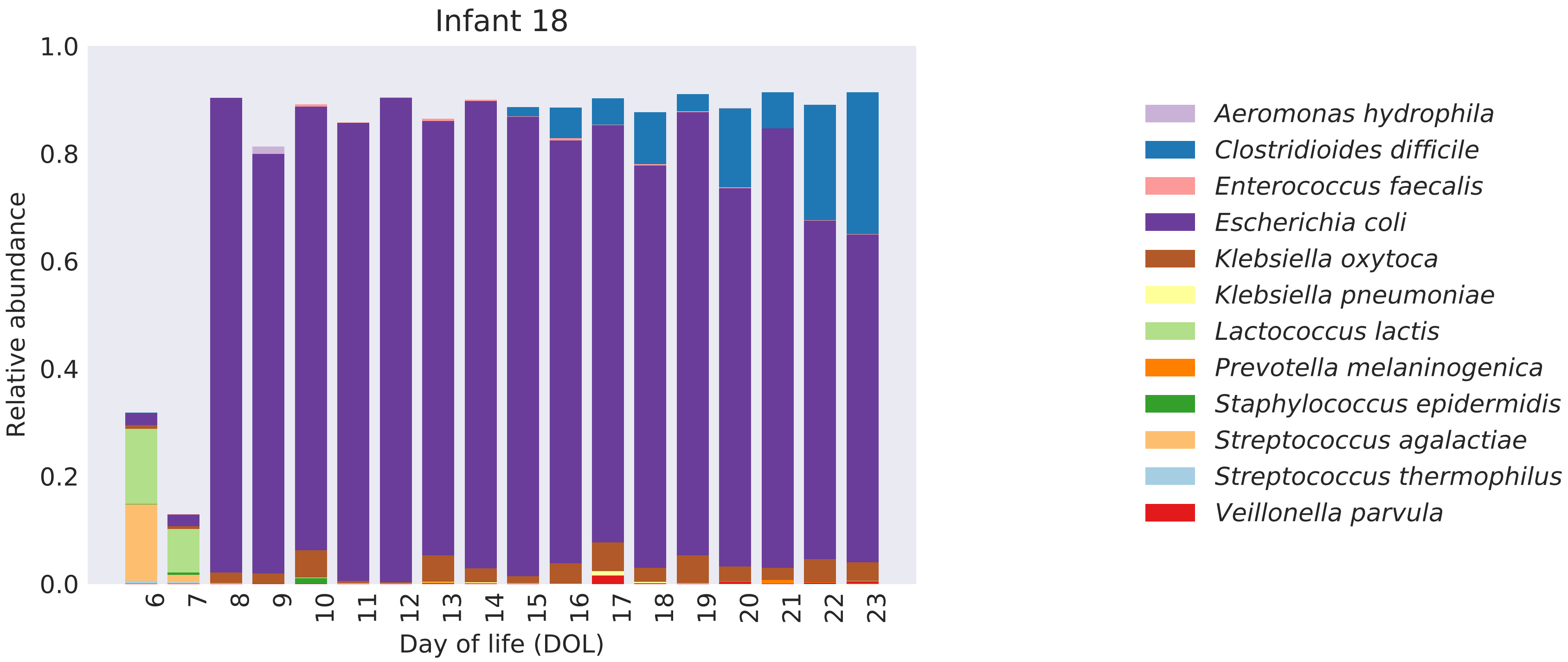
Infant 18
Relative abundance
1.0
0.8
0.6
0.4
0.2
0.0
6
7
8
9
10
11
12
13
14
15
16
17
18
19
20
21
22
23
Day of life (DOL)
Aeromonas hydrophila
Clostridioides difficile
Enterococcus faecalis
Escherichia coli
Klebsiella oxytoca
Klebsiella pneumoniae
Lactococcus lactis
Prevotella melaninogenica
Staphylococcus epidermidis
Streptococcus agalactiae
Streptococcus thermophilus
Veillonella parvula
